# Artificial Intelligence and Machine learning based prediction of resistant and susceptible mutations in *Mycobacterium tuberculosis*

**DOI:** 10.1038/s41598-020-62368-2

**Published:** 2020-03-26

**Authors:** Salma Jamal, Mohd. Khubaib, Rishabh Gangwar, Sonam Grover, Abhinav Grover, Seyed E. Hasnain

**Affiliations:** 1 Jamia Hamdard Institute of Molecular Medicine, Jamia Hamdard, Hamdard Nagar, New Delhi, 110062 India; 2 School of Biotechnology, Jawaharlal Nehru University, New Mehrauli Road, New Delhi, 110 067 India; 3 Dr. Reddy’s Institute of Life Sciences, University of Hyderabad Campus, Professor C.R. Rao Road, Hyderabad, 500046 India

**Keywords:** Computational models, Machine learning

## Abstract

Tuberculosis (TB), an infectious disease caused by *Mycobacterium tuberculosis* (*M.tb*), causes highest number of deaths globally for any bacterial disease necessitating novel diagnosis and treatment strategies. High-throughput sequencing methods generate a large amount of data which could be exploited in determining multi-drug resistant (MDR-TB) associated mutations. The present work is a computational framework that uses artificial intelligence (AI) based machine learning (ML) approaches for predicting resistance in the genes *rpoB*, *inhA*, *katG*, *pncA, gyrA* and *gyrB* for the drugs rifampicin, isoniazid, pyrazinamide and fluoroquinolones. The single nucleotide variations were represented by several sequence and structural features that indicate the influence of mutations on the target protein coded by each gene. We used ML algorithms - naïve bayes, k nearest neighbor, support vector machine, and artificial neural network, to build the prediction models. The classification models had an average accuracy of 85% across all examined genes and were evaluated on an external unseen dataset to demonstrate their application. Further, molecular docking and molecular dynamics simulations were performed for wild type and predicted resistance causing mutant protein and anti-TB drug complexes to study their impact on the conformation of proteins to confirm the observed phenotype.

## Introduction

Tuberculosis (TB) is a contagious disease caused by the bacterium *Mycobacterium tuberculosis* (*M.tb*). According to the latest World Health Organization Report (WHO 2018), 10 million people were infected and 1.6 million died of TB in 2017, which included 0.3 million HIV associated deaths^1^. TB is curable and the current line of treatment includes a combination of four first-line drugs - rifampicin, isoniazid, ethambutol and pyrazinamide. However, the problem is intensified due to the development of multi drug resistance (MDR) due to improper usage of anti-TB medicines, poor quality drugs, non-compliance of treatment regime by the patient, or transmission of resistant strains of *M.tb* or simply activation of drug efflux pumps^2^. MDR-TB is curable by fluoroquinolones, however, these are limited, expensive, toxic and require longer treatment duration^3^. Thus, detection of resistance conferring mutations will help in rapid diagnosis of DR/MDR-TB and understanding the mechanism of resistance to develop effective treatment strategies.

The standard technique for *M.tb* drug susceptibility testing is a culture based method, which compares the growth of the bacteria in the presence and absence of an anti-bacterial drug^4^. The traditional method of phenotypic drug susceptibility testing is challenging due to the delayed detection of resistance owing to slow bacterial growth and poor reproducibility of results in case of most of the drugs^5^. As the problem of drug resistance has intensified, various high-throughput sequencing methods and genotyping techniques have been developed to identify the resistance conferring mutations^6–8^. The introduction of next-generation sequencing techniques has led to an enormous amount of data^9^. However, the sensitivity of these methods varies depending on the drug resulting in misclassifications. Also, majority of these methods only detect frequently occurring mutations^2,10^.

The extensive data available in the public domain could be exploited for the efficient and accurate identification of resistance causing mutations. It has already been well established that the primary cause of *M.tb* resistance are mutations in genes encoding specific target proteins^8,11^. Thus, the need of the hour is a rapid method that can detect the mutations responsible for drug resistance from the gene sequence.

Machine learning (ML) techniques have been successfully used for building predictive classification models including the identification of compounds based on their biological activities^12,13^, side effect predictions^14,15^, novel disease-associated gene prediction^16^, microarray data analysis^17^, drug discovery against TB^18,19^ and many more^20–22^. AI based ML learns from the known features of data and then makes predictions on blind data^23^. In the present study, Artificial Intelligence (AI) and ML algorithms were used to classify single nucleotide variations (SNVs) as being resistant or susceptible in TB and predict novel resistance conferring mutations. In this work four ML algorithms, naive Bayes (NB), k nearest neighbor (kNN), support vector machine (SVM), and artificial neural network (ANN), were used for the prediction task. Several mutations have been identified which may cause drug resistance in *M.tb*. Various sequence and structure based features were used to capture the impact of these mutations for each target gene. Additionally, a feature selection method was used to identify the features having the most significant role in classifying a mutation as susceptible or resistant. Molecular docking along with molecular dynamics (MD) simulations studies were performed for wild type and mutant, predicted to be resistance causing, protein-drug complexes to analyze the effect of the mutations. The present study describes an integrative computational approach to generate AI and ML based models using the various sequence and structural features of SNVs in *M.tb* genes for the prediction of resistance conferring mutations.

## Results

### Dataset preparation

Single nucleotide variations were obtained for *rpoB*, *inhA*, katG, *pncA*, *gyrA*, and *gyrB*. Machine learning models were generated only for mutations associated with proteins having an experimental structure available in the protein data bank (PDB). The total number of variations obtained from the TBDReaMDB and GMTV databases for each TB drug and the number of mutations obtained after data preprocessing have been provided in Table 1. The number of genes/mutations included in the final training dataset and testing dataset, and the actual number of resistant and susceptible mutations included in both training and test datasets have been provided in Table 2. The final datasets of the variations and descriptors used for model generation have been provided in Supplementary Tables S1–S6.

**Table 1:** Total number of variations obtained from TBDReaMDB and GMTV database for each TB drug and the number of mutations obtained after data preprocessing.

Drug	Gene	TBDReaMDB	GMTV	Final variations
Rifampin	*rpoB*	134	198	114
Isoniazid	*InhA*	13	30	27
	*katG*	273	83	250
Pyrazinamide	*pncA*	278	137	241
Fluoroquinolones	*gyrA*	17	112	73
	*gyrB*	18	72	49

**Table 2:** Number of genes/mutations included in the final training dataset and testing dataset, and the actual number of resistant and susceptible mutations included in both training and test dataset.

Drug	Gene	Training set			Testing set		
		Resistant	Susceptible	Total	Resistant	Susceptible	Total
Rifampin	*rpoB*	40	52	92	10	12	22
Isoniazid	*InhA*	8	14	22	2	3	5
	*katG*	108	92	201	27	23	50
Pyrazinamide	*pncA*	112	81	193	27	21	48
Fluoroquinolones	*gyrA*	25	33	58	6	8	14
	*gyrB*	23	17	40	5	4	9

### Model evaluation and comparison of machine learning algorithms

**Figure 1:**
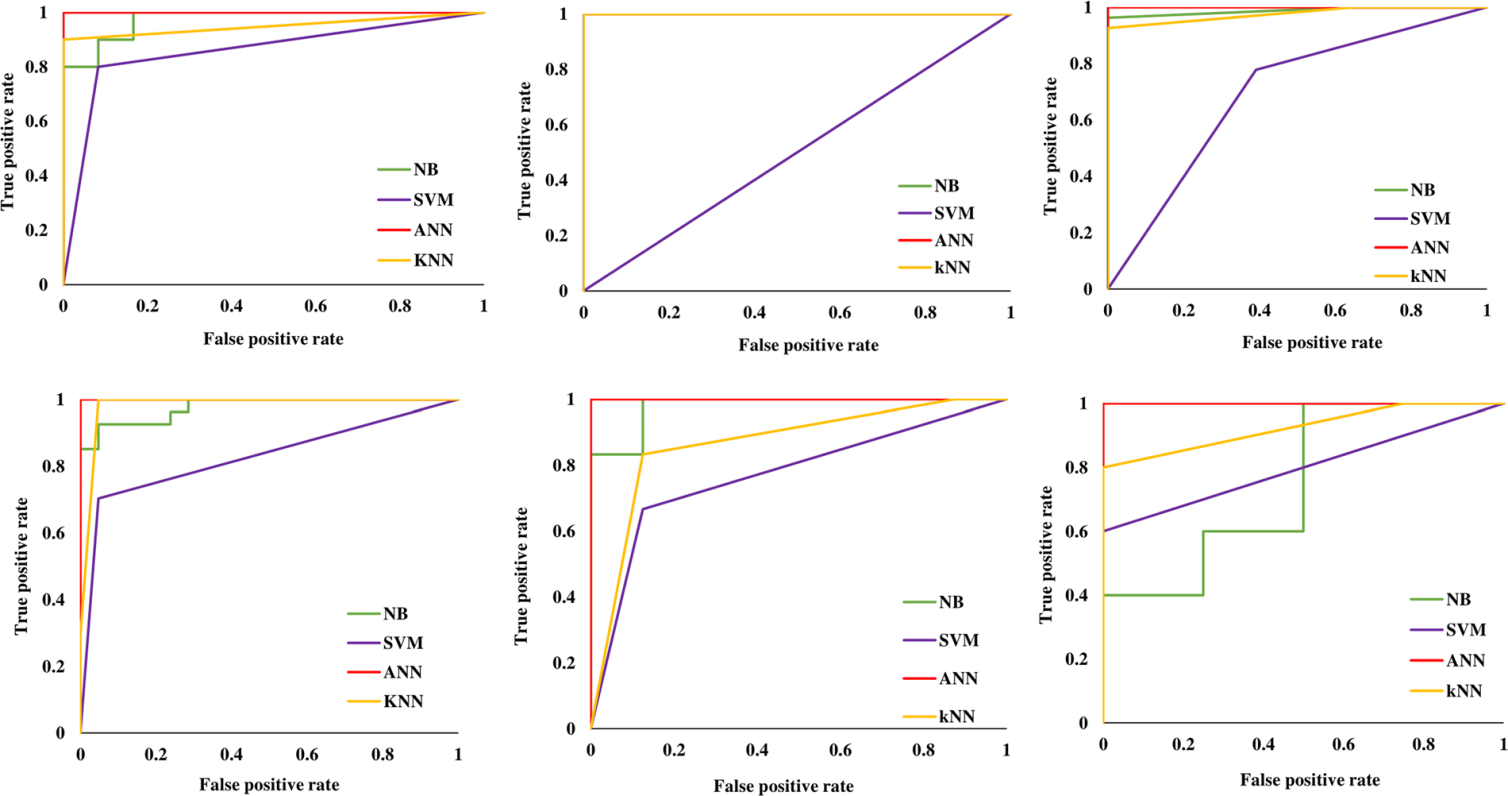
ROC plots for all the models generated for genes (**A**) *rpoB*, (**B**) *pncA*, (**C**) *inhA*, (**D**) *katG*, (**E**) *gyrA* and (**F**) *gyrB*.

The performance of the classification models on the training data set using 10-fold cross validation is summarized in Table 3. All AI/ML models for the genes had good overall accuracy of approximately 70%. ANN performed the best with the highest accuracy models for most genes in the 10-fold cross validation. In the non-redundant testing data, we were able to categorize mutations as susceptible or resistant with an accuracy ranging between 66.66–100%. (Table 4). The ANN models had the overall best performance for testing data, followed by the kNN models with high accuracies and AUC values. The ROC plots for all the models are shown in Fig. 1.

**Table 3:** The performance of the classification models on the training data set using 10-folds cross validation.

Gene	Measure/Methods	NB	SVM	ANN	kNN
*rpoB*	Accuracy	88.04%	84.78%	95.65%	86.95%
	AUC	0.92	0.83	0.99	0.87
*InhA*	Accuracy	90.90%	63.63%	95.45%	90.90%
	AUC	0.75	0.5	1	0.9
*katG*	Accuracy	84%	78.50%	98.50%	92%
	AUC	0.94	0.77	0.99	0.91
*pncA*	Accuracy	83.93%	75.12%	99.48%	90.67%
	AUC	0.96	0.76	1	0.9
*gyrA*	Accuracy	75.86%	72.41%	86.20%	81.03%
	AUC	0.86	0.71	0.95	0.82
*gyrB*	Accuracy	82.50%	70%	97.50%	97.50%
	AUC	0.91	0.68	0.96	0.97

**Table 4:** The performance of the classification models on the non-redundant testing data set.

Gene	Measure/Methods	NB	SVM	ANN	kNN
*rpoB*	Accuracy	90.90%	86.36%	90.90%	95.45%
	AUC	0.97	0.85	1	0.95
*InhA*	Accuracy	100%	60%	81.81%	100%
	AUC	1	0.5	0.92	1
*katG*	Accuracy	98%	70%	98%	96%
	AUC	0.98	0.69	1	0.97
*pncA*	Accuracy	93.75%	81.25%	97.91%	97.91%
	AUC	0.97	0.82	1	0.98
*gyrA*	Accuracy	92.85%	78.57%	100%	85.71%
	AUC	0.97	0.77	1	0.86
*gyrB*	Accuracy	66.66%	77.77%	88.88%	88.88%
	AUC	0.75	0.8	1	0.92

The ANN models gave the best predictions in the case of four genes, *katG*, *pncA*, *gyrA*, *and gyrB*. The kNN models performed the best for four genes, *rpoB*, *inhA*, *pncA*, *and gyrB*, amongst the six genes for which models were built. The NB classifier had the best accuracy for *inhA* and *katG* genes. The AI/ML algorithms in the present study have successfully been used for predicting the effect of mutations on the stability of protein^24–26^. Supplementary Dataset 1 provides the AI/ML models generated in the present study.

### Predictions on non-redundant blind testing data and descriptor selection

The non-redundant blind dataset consisted of the mutations not present in the 80% training dataset. This was the 20% of data not used to train the models and kept separate to evaluate the performance of the predictive models. Feature selection techniques have commonly been used to find important features and remove features of less importance which contribute less to classification. In the present study eight feature selection techniques were used to identify the features contributing to the prediction task: symmetrical uncertainty based selection, relief attribute evaluation, oneR classifier algorithm, gain ratio and info gain based feature selection, bestfirst attribute evaluation, classifier algorithm based selection using zeroR classification algorithm, and correlation based feature selection.

### Rifampicin (rpoB gene)

Rifampicin is a first-line antibiotic used for the treatment of TB. The drug binds to the β-subunit of bacterial RNA polymerase coded by the *rpoB* gene. In *rpoB*, the binding site for rifampicin is the region between amino acids 424–456, which is also known as the rifampicin resistance determining region (RRDR) as the majority of mutations occur here^8,27^. Our models predicted the mutations to be susceptible or resistant with very high accuracy, 95.45% for the kNN model and 90.90% for the NB and ANN models. The mutations outside the RRDR region, which were part of the testing dataset Val490Leu, Phe511Leu and Thr514Ser, were predicted to be resistance conferring by our models. For feature selection, ΔΔG was ranked highest by four of the seven feature selection techniques, thus indicating its important role in the classification of mutations as benign or susceptible. The other top ranked features included residue type (wt), residue type (mutant), and hydrophobicity.

### Isoniazid (inhA and katG genes)

Resistance to the drug isoniazid is primarily due to mutations in the *katG* and *inhA* genes^28^. In *katG*, isoniazid binds to the amino acid residues in the range 104–381, which constitutes the heme-binding channel of the protein^29^. The NB and ANN models performed the best with 98% accuracy for *katG* gene. The mutations in the blind test set predicted to be resistance associated were Ala61Thr, Val68Gly, Ala109Thr, Ala122Val, Phe129Leu, Leu148Arg, Thr180Lys, Trp191Arg, Glu195Lys, Gln224Glu, Val230Ala, Gly234Arg, Thr275Ser, Gly299Ala, Gly299Ser, Pro280His, Tyr304Ser, Gly307Glu, Gly309Cys, Glu318Lys, Trp321Cys, and Ala350Ser. The novel resistance conferring mutations included Asn238Lys, Leu587Ile, Leu619Pro, and Leu634Phe.

In the case of *inhA*, the drug binds to the amino acid residues in the range 14–197^30^ and three of the four models predicted the test set mutations Ile16Thr and Ala124Glu as resistance conferring. The NB model had the highest accuracy and AUC value with 100% accuracy and 1.00, respectively.

For both *katG* and *inhA* genes, ΔΔG was ranked highest by most of the feature selection algorithms. In the case of *katG*, polarity, residue type (wt), residue type (mutant), and hydrophobicity were among the other significant contributing features. Accessible surface area (ASA) also played an important role and was ranked fourth by four feature selection algorithms, uncertainty based attribute evaluation, relief attribute evaluation, oneR algorithm, and the classifier algorithm using the zeroR classifier. In the case of *inhA*, ΔΔG again ranked highest followed by residue type (mutant) as features primarily contributing to the mutation prediction task. Hydrophobicity and polarity are the other top ranked features along with the secondary structure feature, which was ranked fifth among the top five by uncertainty based, gain ratio and info gain algorithms, and correlation based feature selection algorithms.

### Pyrazinamide (pncA gene)

Mutations in the *pncA* gene, which alter the binding of the drug pyrazinamide, are considered the major cause of resistance in *M.tb*. The mutations in this case are reported to be scattered throughout the *pncA* gene^27^. The models kNN and ANN had the best accuracy, 97.91% and an AUC value of 0.97 and 1.00, respectively. The novel mutations predicted to confer resistance to the drug by the models included Cys96Glu and Val155Met. The top ranked feature having the greatest impact on the prediction task was ΔΔG, followed by residue type (mutant), residue type (wt), polarity, and hydrophobicity.

### Fluoroquinolones (gyrA and gyrB genes)

Fluoroquinolones are a group of antibiotics used to treat bacterial infections and have been an attractive treatment strategy in case of MDR-TB^31^. The target genes for fluoroquinolones (*gyrA* and *gyrB)* and mutations in the quinolone resistance determining regions (QRDR) of these genes are strongly related to drug resistance^32^. The ANN models performed best for both genes, *gyrA* and *gyrB*, with accuracies of 100% and 88.88% individually and AUC values of 1.00. The novel mutations predicted to cause resistance by the models included Gln431Glu and Leu711Met in case of *gyrA* and Asn499Thr in case of *gyrB*.

For *gyrA*, ΔΔG had the highest correlation and was ranked as the topmost contributing feature followed by residue type (wt), residue type (mutant), polarity, and ASA. Molecular weight was identified as an important feature for classification by correlation based feature selection, and also ranking highest using the best first feature selection method. In the case of *gyrB*, ΔΔG was again the feature with greatest influence on the prediction task. Other features included residue type (wt), isoelectric point, hydrophobicity, and volume. Supplementary Tables S7–S12 provides the ranking of features selected by various feature selection techniques for all the genes, *rpoB*, *pncA*, *inhA*, *katG*, *gyrA*, and *gyrB*.

### Impact of predicted resistance associated mutations on drug binding

**Figure 2:**
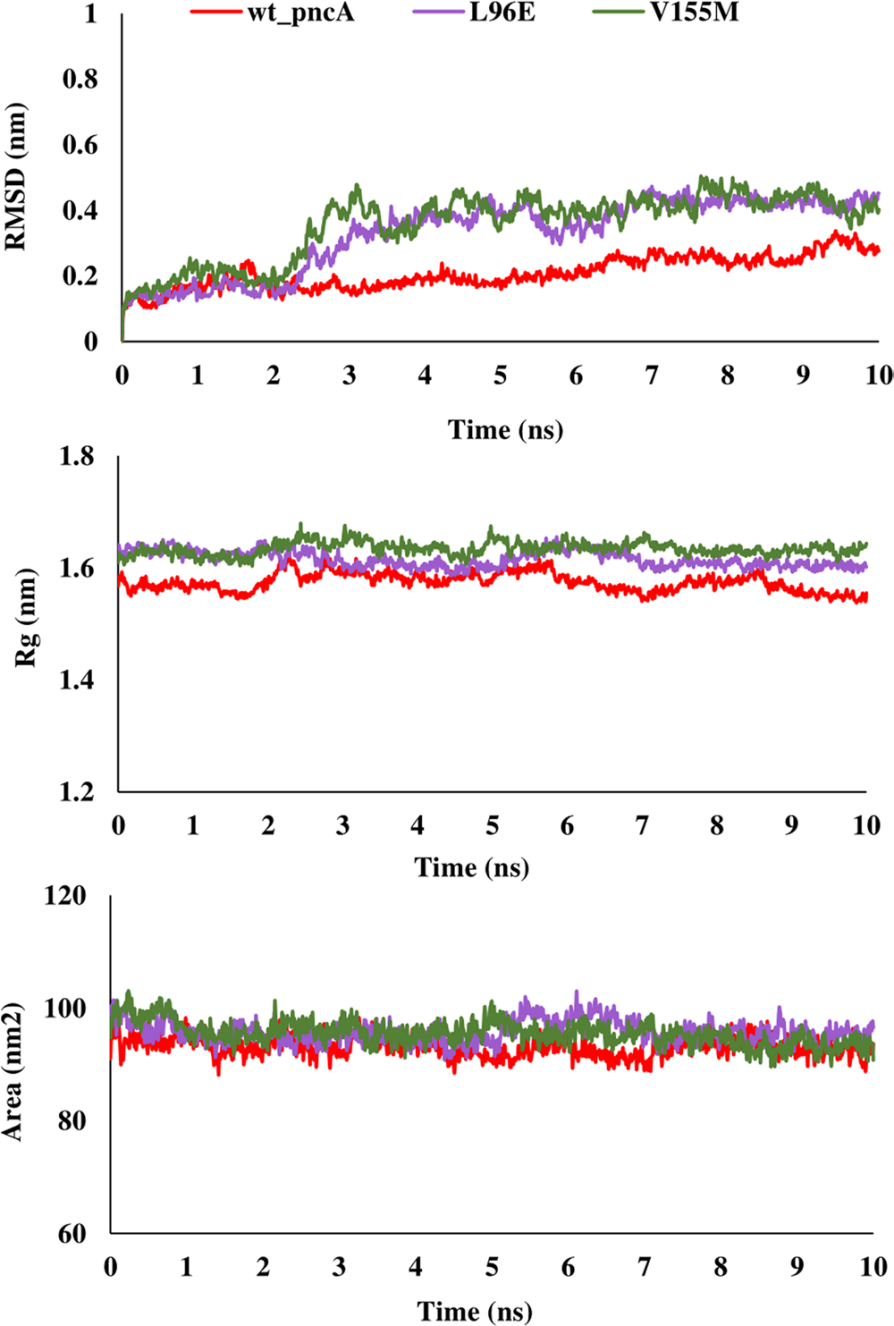
RMSD, Rg and SASA plot for *pncA* gene. The RMSD, Rg and SASA were less in case of wild type indicating that the mutations destabilized the protein.

**Figure 3:**
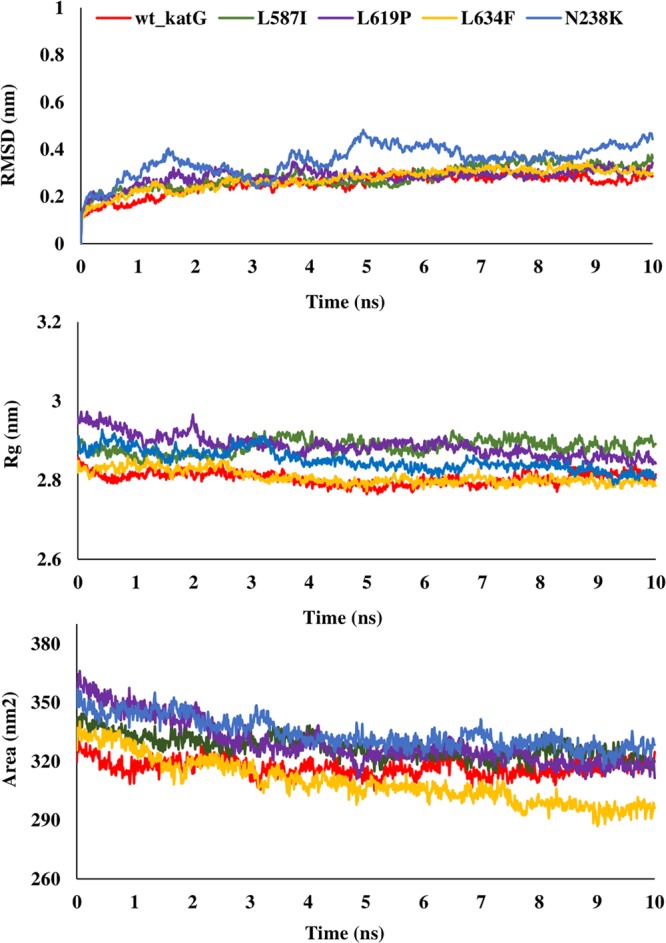
RMSD, Rg and SASA plot for *katG* gene. The RMSD, Rg and SASA of mutants were higher that wild type demonstrating that the wild type protein was more stable.

**Figure 4:**
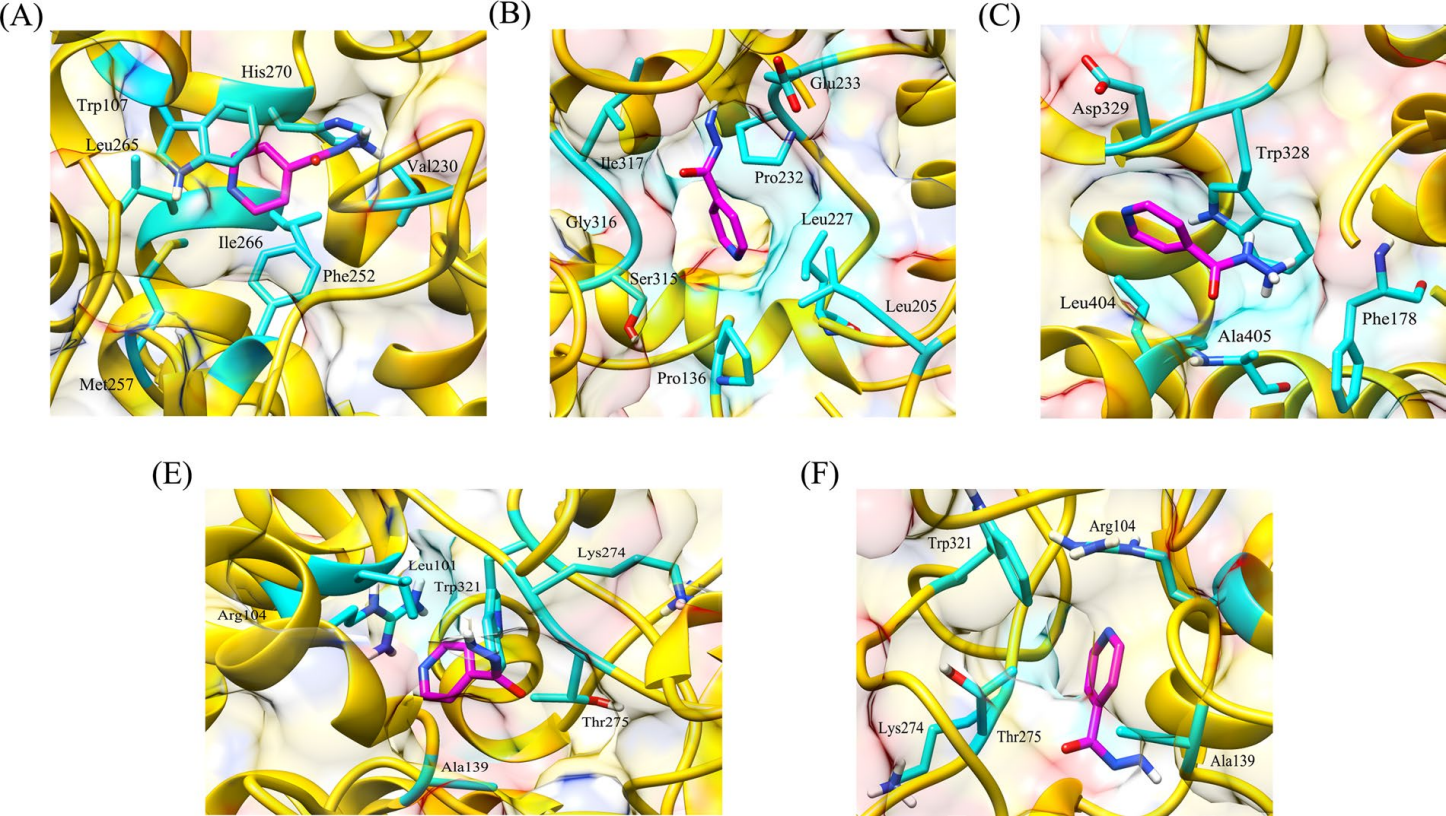
Interaction patterns between (**A**) wild type and (**B**) L587I (C) L619P (**D**) L634F (**E**) N238K mutant protein-isoniazid complexes. The drug bound to protein through hydrophobic interactions only, however strong binding was observed in wild type protein.

The binding free energy of mutant and wild type drug bound proteins was calculated using Schrodinger Glide docking. It was observed that the energy for mutant proteins was quite less than wild-type. Table 5 provides the docking scores of wild type and mutant protein-drug complexes along with their pre-MD interactions. To study the diverse impact of mutations on protein, MD simulations studies were performed on the docked conformations of protein-drug complexes. RMSD was calculated to ensure the stability of the system over the entire simulation run. The RMSD plots for all the proteins during the entire simulation run indicated the stability of the protein and that they can be considered for further analyses. The RMSD for wild type and mutant catalase-peroxidase (*katG*), pyrazinamidase (*pncA*), gyrase A (*gyrA*) and gyrase B (*gyrB*) proteins, was found to fluctuate between 0.1–0.4 Å. The radius of gyration (Rg) is the degree of compactness of the protein, and solvent accessible surface area (SASA) is the measure of the residues exposed to the surface. In the case of the *katG* and *pncA* genes, the Rg and SASA for mutant models was remarkably higher in comparison to wild type proteins, which are clearly visible in Figs. 2 and 3, respectively. This indicated that the wild type proteins were more stable, compactly packed and buried inside the core than the mutants (Table 6). The interaction patterns between wild type and mutant protein-drugs complexes were also in line with MD trajectory analysis as is evident from Figs. 4 (*katG*) and 5 (*pncA*). No hydrogen bonding interactions were observed in protein-isoniazid complex for both wild type and mutants suggesting that the hydrophobic interactions were the main stabilizing interactions in both the cases, however strong binding was observed in wild type protein. The number of residues forming hydrogen bonds was two in case of wild type protein-pyrazinamide complex and L96E mutation and one for V155M mutations. Fewer interacting residues were observed in case of mutants in comparison to wild type pointing towards more binding affinity in the latter case.

**Table 5:** Docking scores of wild-type and mutant drug bound proteins.

Gene	Drug	Wild type and mutants	Glide docking score (kcal/mol)
*katG*	Isoniazid	wild type	−4.41
		L587I	−4.29
		N238K	−4.09
		L634F	−4.30
		L619P	−4.17
*pncA*	Pyrazinamide	wild type	−4.20
		L96E	−3.55
		V155M	−3.48
Fluoroquinolones			
*gyrA* (N-terminal)		wild type	
	Ofloxacin		−3.18
	Moxifloxacin		−2.17
	Ciprofloxacin		−2.86
		L711M	
	Ofloxacin		−1.14
	Moxifloxacin		−0.09
	Ciprofloxacin		−2.39
*gyrA* (C-terminal)		wild type	
	Ofloxacin		−2.72
	Moxifloxacin		−3.00
	Ciprofloxacin		−3.52
		Q431E	
	Ofloxacin		−2.34
	Moxifloxacin		−2.24
	Ciprofloxacin		−2.85
*gyrB*		wild type	
	Ofloxacin		−4.48
	Moxifloxacin		−4.15
	Ciprofloxacin		−4.07
		N499T	
	Ofloxacin		−3.86
	Moxifloxacin		−3.79
	Ciprofloxacin		−2.05

**Table 6:** Average values of RMSD, Rg and SASA for wild type and mutant protein-drug complexes over the course of entire MD simulations run.

Gene	Drug	Wild type and mutants	RMSD (nm)	Rg (nm)	SASA(nm^2^)
*katG*	Isoniazid	wild type	0.25	2.80	316.90
		L587I	0.28	2.88	327.19
		N238K	0.28	2.88	325.70
		L634F	0.27	2.80	309.39
		L619P	0.35	2.84	334.17
*pncA*	Pyrazinamide	wild type	0.20	1.57	93.10
		L96E	0.33	1.61	95.73
		V155M	0.35	1.63	95.49
Fluoroquinolones					
*gyrA* (N-terminal)		wild type			
	Ofloxacin		0.18	1.97	157.29
	Moxifloxacin		0.18	1.94	155.76
	Ciprofloxacin		0.18	1.93	154.25
		L711M			
	Ofloxacin		0.20	1.92	153.61
	Moxifloxacin		0.18	1.92	152.33
	Ciprofloxacin		0.19	1.92	153.59
*gyrA* (C-terminal)		wild type			
	Ofloxacin		0.28	2.95	270.43
	Moxifloxacin		0.27	2.95	268.73
	Ciprofloxacin		0.27	2.96	269.43
		Q431E			
	Ofloxacin		0.29	2.97	267.41
	Moxifloxacin		0.24	7.27	259.24
	Ciprofloxacin		0.26	2.98	262.34
*gyrB*		wild type			
	Ofloxacin		0.19	1.96	142.37
	Moxifloxacin		0.22	1.93	144.99
	Ciprofloxacin		0.26	1.96	144.32
		N499T			
	Ofloxacin		0.23	1.94	144.17
	Moxifloxacin		0.28	1.95	144.61
	Ciprofloxacin		0.20	1.93	142.66

**Figure 5:**
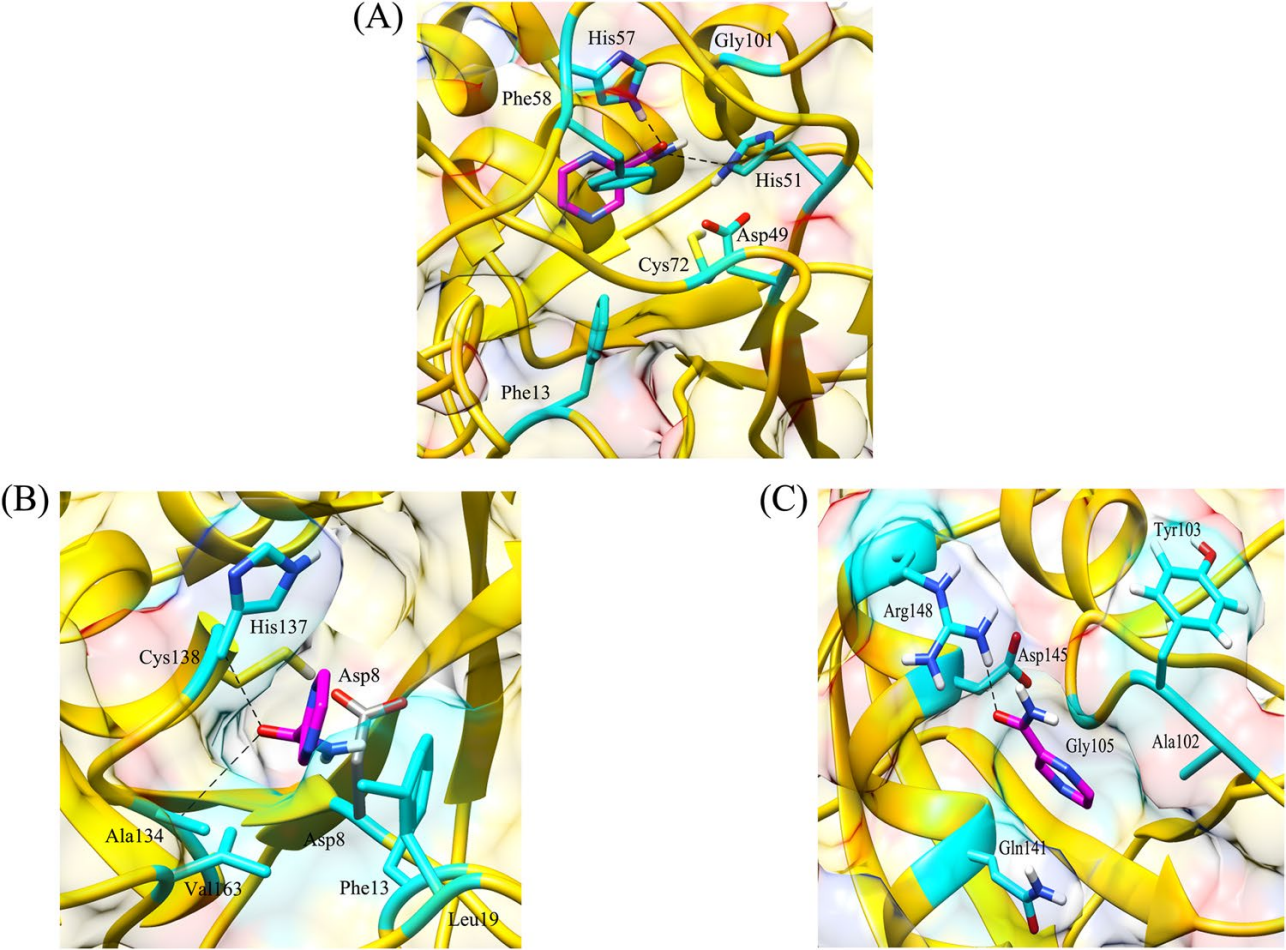
Hydrogen bonding and hydrophobic interactions seen in (**A**) wild type, (**B**) L96E and (**C**) V155M mutant protein-pyrazinamide complexes. Fewer interacting residues were observed in case of mutants in comparison to wild type.

**Figure 6:**
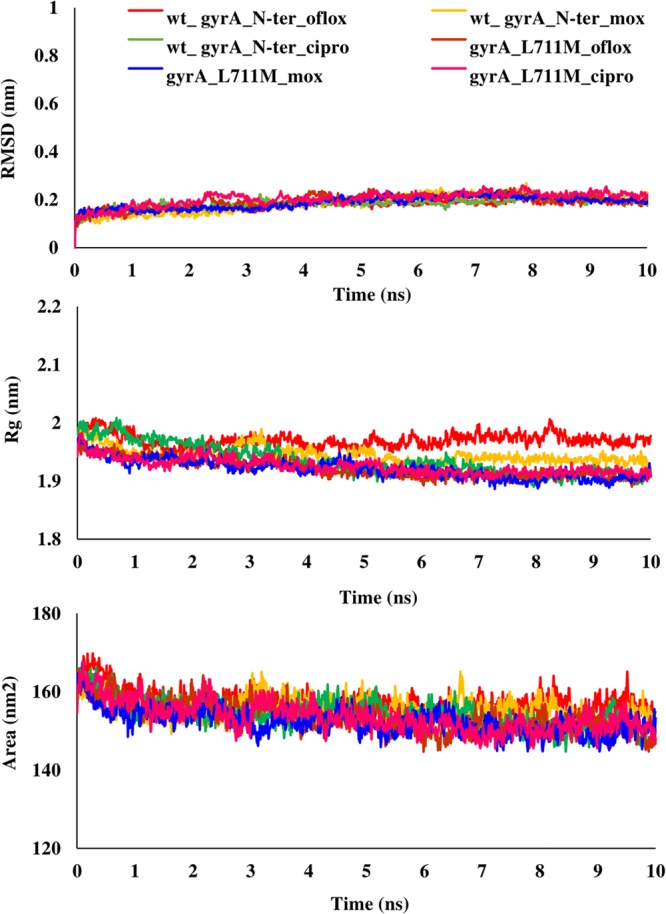
RMSD, Rg and SASA plot for *gyrA* gene, N-terminal protein. The plots for RMSD, Rg and SASA were similar to wild type in case of mutant, L711M.

**Figure 7:**
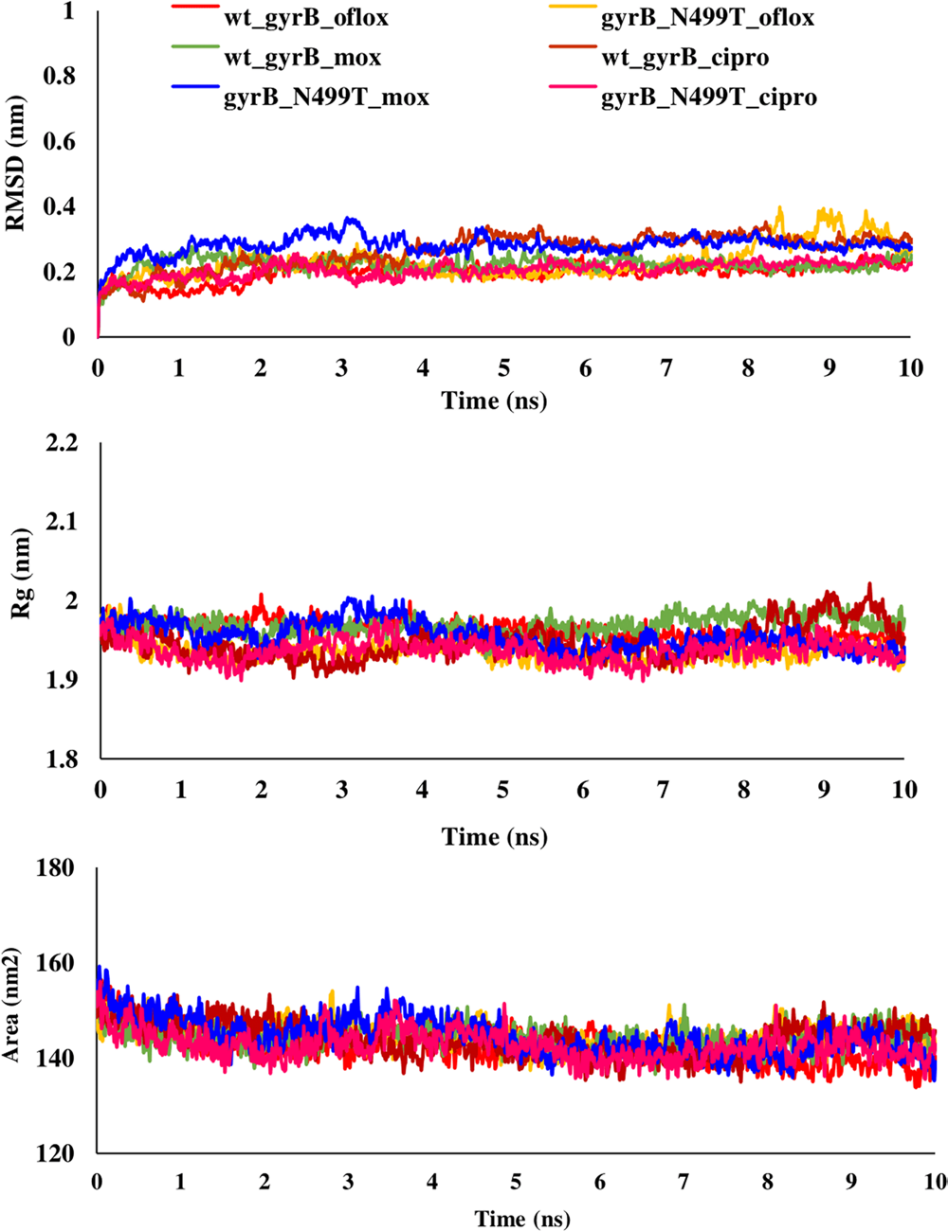
RMSD, Rg and SASA plot for *gyrA* gene, C-terminal protein. For Q431E mutant, the RMSD and Rg were slightly higher than wild type, however SASA was less for mutant protein.

**Figure 8:**
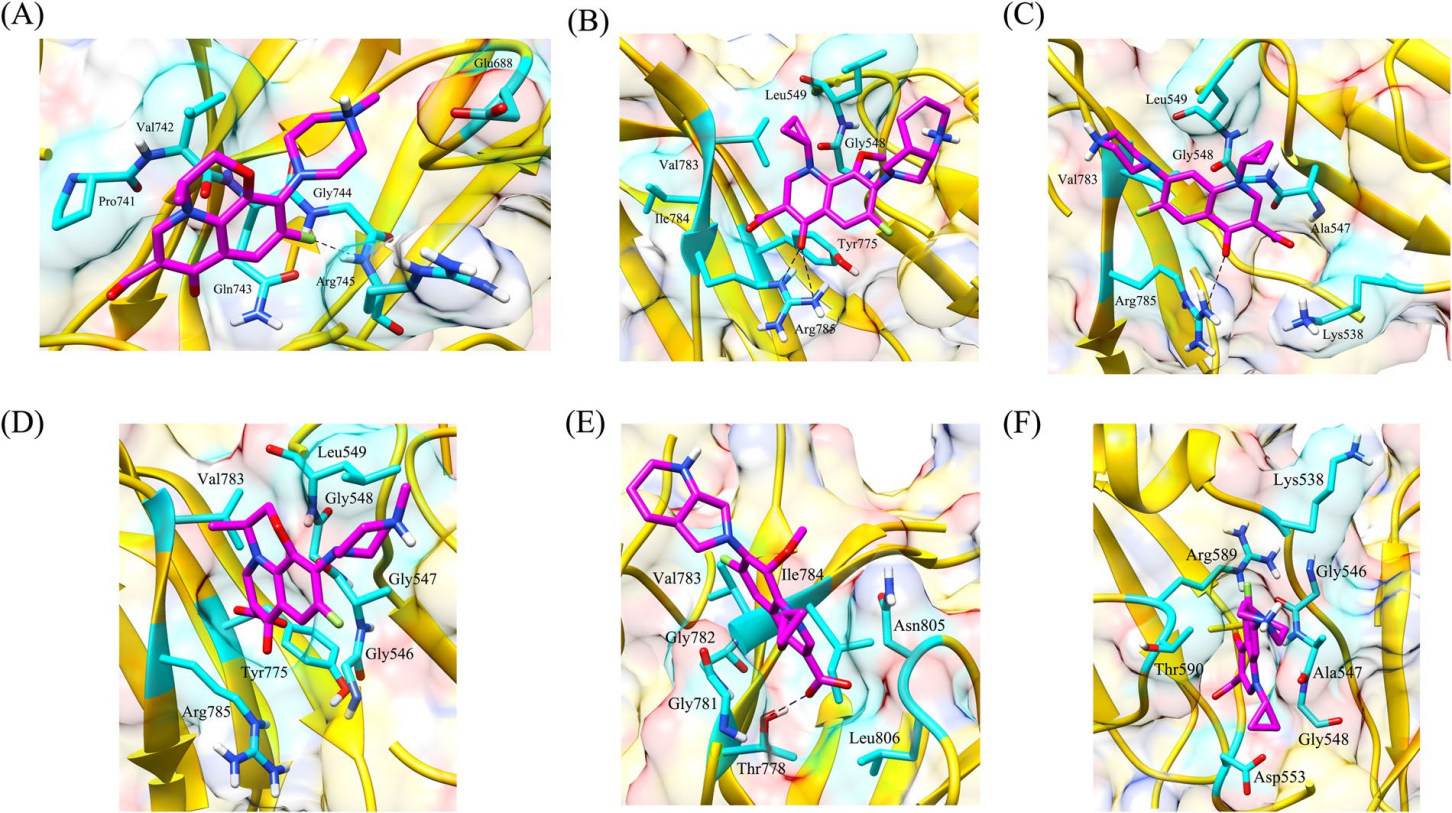
Interaction pattern observed between N-terminal of wild type gyrase A and fluoroquinolones; (**A**) ofloxacin; (**B**) moxifloxacin; (**C**) ciprofloxacin and mutant, L711M; (**D**) ofloxacin; (**E**) moxifloxacin and (**F**) ciprofloxacin. The wild type protein formed hydrogen bonds with the drugs whereas no hydrogen bond was present in case of mutant protein-drug complexes.

**Figure 9:**
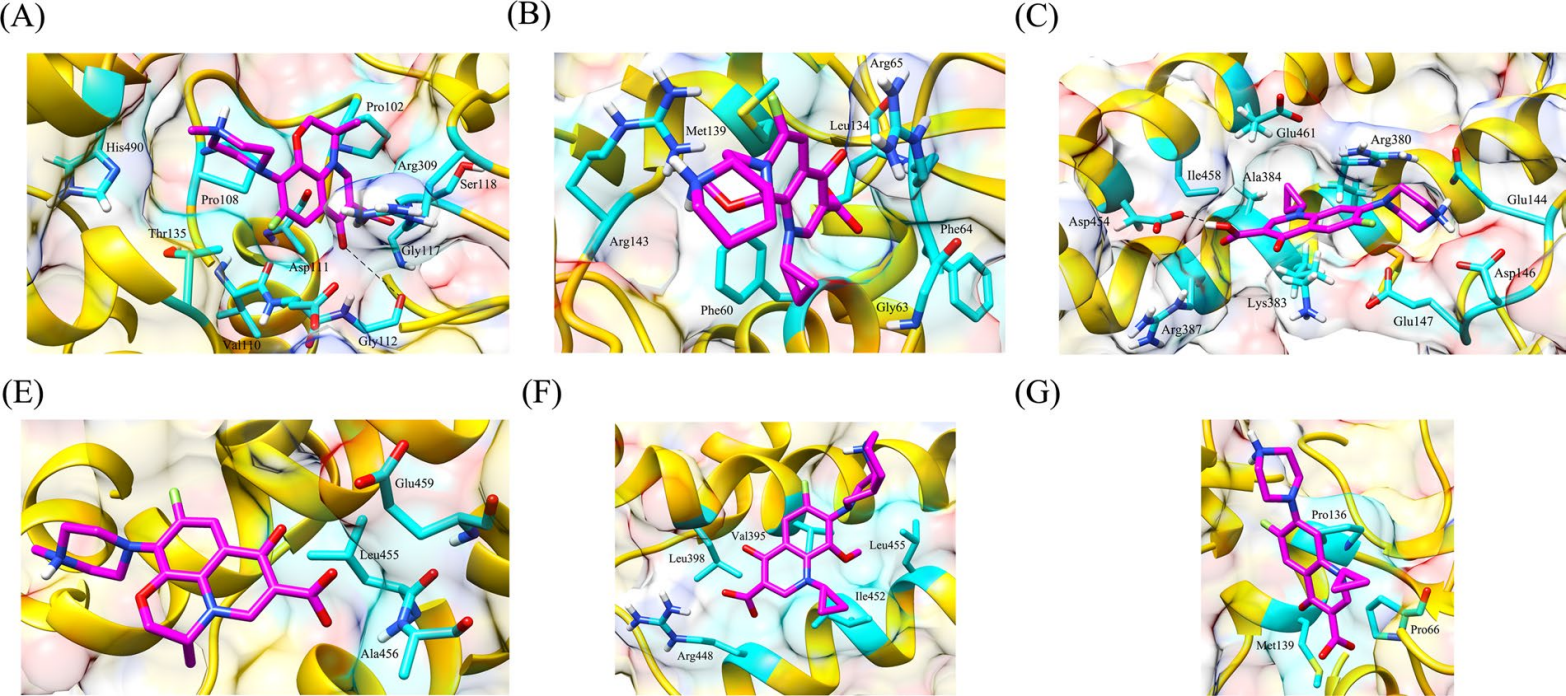
Interaction pattern observed between C-terminal of wild type gyrase A and fluoroquinolones; (**A**) ofloxacin; (**B**) moxifloxacin; (**C**) ciprofloxacin and mutant, Q431E; (**D**) ofloxacin; (**E**) moxifloxacin and (**F**) ciprofloxacin. More number of interacting residues were present in wild type protein bound to the drugs than in mutant protein-drug complexes.

**Figure 10:**
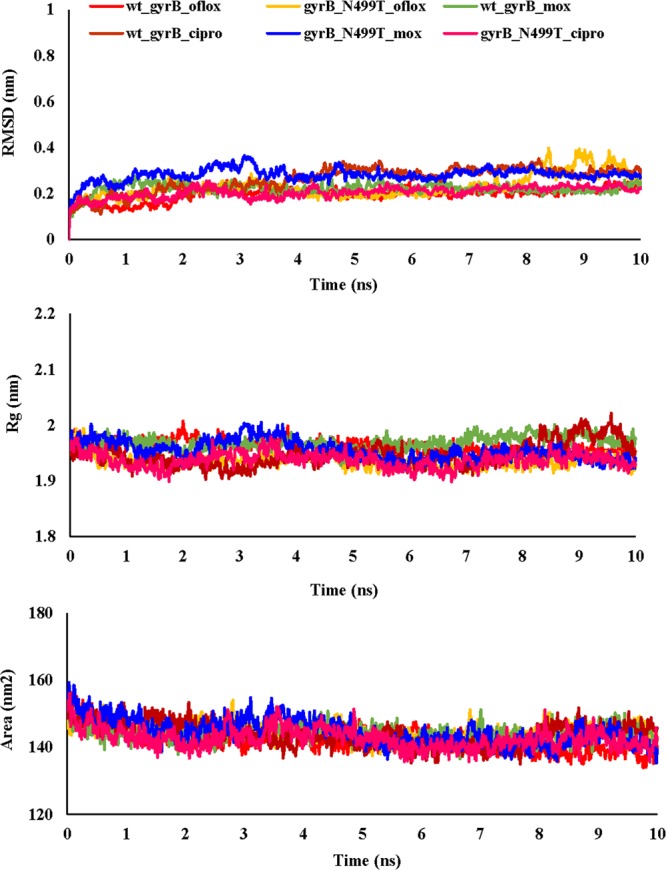
RMSD, Rg and SASA plot for *gyrB* gene. The RMSD was higher for mutant while Rg and SASA were approximately similar for wild type and mutant showing that mutation did not had much impact on the protein.

**Figure 11:**
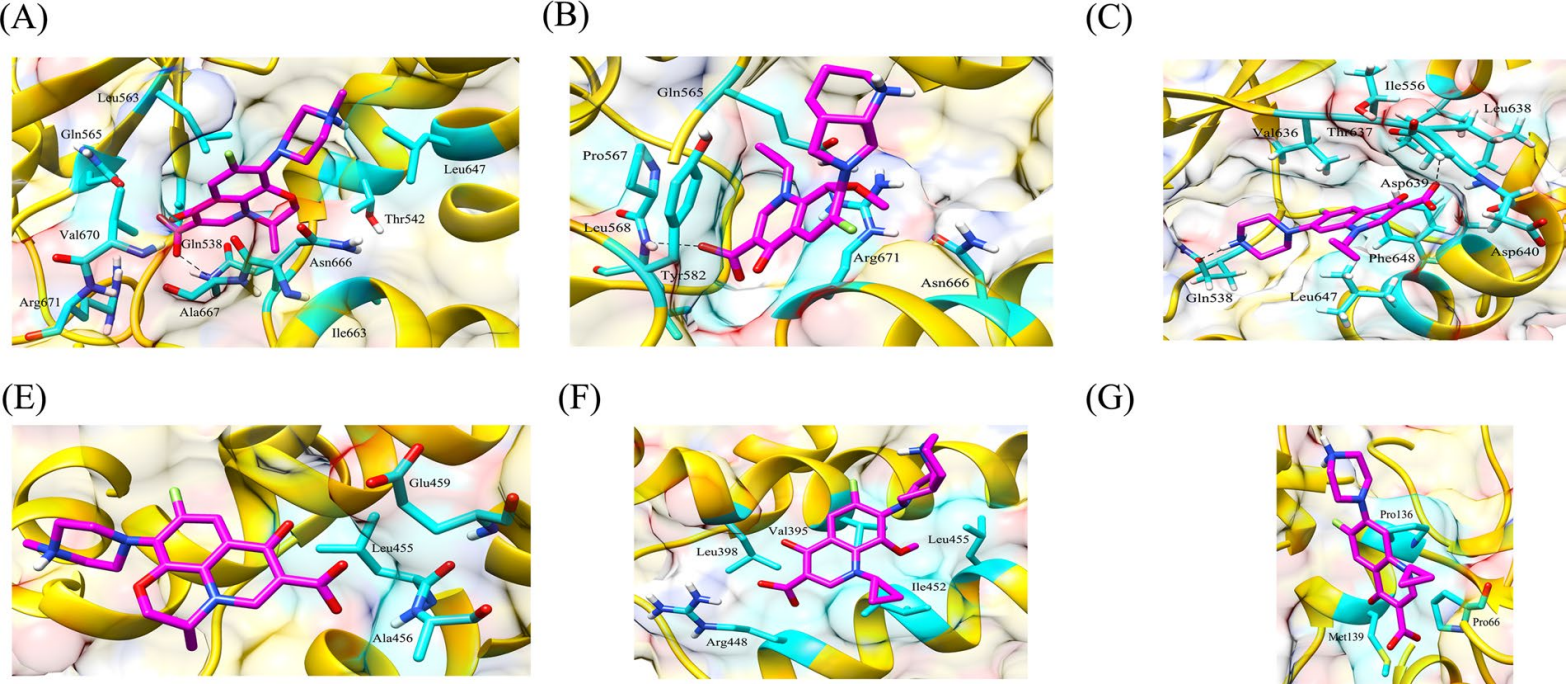
Hydrogen bonding and hydrophobic interactions between wild type gyrase B and various drugs (**A**) ofloxacin; (**B**) moxifloxacin; (**C**) ciprofloxacin and mutant protein, N499T; (**D**) ofloxacin (**E**) moxifloxacin and (**F**) ciprofloxacin. In case of mutant proteins, only weak hydrophobic interactions were seen.

Talking about the complex of proteins with fluoroquinolones, similar RMSD, Rg, and SASA plots were obtained for wild type and L711M mutant in gyrase A N-terminal. The RMSD and Rg values were marginally higher in mutant, Q431E as compared to wild type gyrase A C-terminal. RMSD, Rg and SASA plots for the wild type and mutants in gyrase A N- and C-terminal are shown in Figs. 6 and 7. All the three fluoroquinolones formed hydrogen bonds, ofloxacin (one), moxifloxacin (two) and ciprofloxacin (one), however no hydrogen bonds were seen for mutation, L711M, except for moxifloxacin (one) occurring in gyrase A N-terminal (Fig. 8). Talking about C-terminal of gyrase A (Fig. 9), the wild type protein formed one hydrogen bond with ofloxacin whereas the wild type protein with moxifloxacin and ciprofloxacin was bounded to ligand by hydrophobic interactions. For the mutation Q431E, falling in C-terminal, hydrophobic interactions were observed but less in number in comparison to wild type suggesting more stable complex for latter. As evident in Fig. 10, the RMSD of wild type was less in gyrase B (except in case of ciprofloxacin) than the mutant models, however the Rg and SASA values were almost similar. The hydrogen bonding and hydrophobic interactions between wild type and mutant protein and drugs in case of gyrase B have been illustrated in Fig. 11, respectively. The wild type gyrase B protein formed one hydrogen bond with each drug which was lost in N499T mutation and only weak hydrophobic interactions were seen. All these results revealed that the resistance conferring mutations destabilized the protein in case of gyrase A and B but to a less extent (Table 6).

### External dataset validation

To demonstrate the real applications of the predictive classification models generated in the present study, the models were evaluated on a blind testing dataset. This testing dataset contained mutations obtained from the MUBII-TB-DB^33^ database, which includes a set of *M.tb* mutations associated with *rpoB*, *pncA*, *inhA*, *katG*, *gyrA*, *gyrB*, and *rrs*. The database contains the resistance data from the TBDReaMDB database as well as studies published before 2013. A total of 130, 237, 11, 263, 17, and 16 variations were obtained for *rpoB*, *pncA*, *inhA*, *katG*, *gyrA*, and *gyrB*, respectively. Prior to testing, the dataset was made non-redundant by removing the mutations that were part of the training or testing dataset used for model generation and validation, respectively. Post-processing the number of final mutations for which predictions were made were 97 for *rpoB*, 38 for *pncA*, 11 for *inhA*, 197 for *katG*, 17 for *gyrA*, and 16 for *gyrB*, respectively. The mutations were considered resistance causing if they were predicted to be resistant by all four methods (NB, ANN, SVM, and kNN), or by at least three of the four methods. A total of 21, 10, 5, 89, 2 and 4 mutations each for *rpoB, pncA, inhA, katG, gyrA* and *gyrB* were predicted to be resistance conferring as listed in Table 7.

**Table 7:** Mutations from external blind dataset predicted to be resistance causing by our models.

Gene	Mutations
*rpoB*	F430S, G432D, G432S, S434R, Q435K, L436R, S437R, Q438K, Q438R, D441E, D441N, N444K, L449S, H451D, H451N, H451Q, H451R, H451S, H451T, P460S, I486T
*InhA*	I16T, I21T, I47T, I95T, I194T
*katG*	L48Q, A61T, A65T, A66P, I71N, M84I, Q88R, G99E, A106V, W107R, H108D, H108E, H108Q, A109V, A110V, G121V, M126I, A139P, L148R, Y155S, A162T, G169A, A172T, A172V, M176I, G186V, W191R, G234E, G234R, A243S, M257T, M257I, T262R, A264T, G279D, A281V, G285D, A291P, G297V, G299A, W300G, Y304S, G305A, G307R, G307A, G307E, G309S, G309D, G316S, G316D, W321R, W321L, W321S, W328G, W328L, W328S, I335T, L336R, W341S, A350T, A350S, A361D, A379V, L384R, I393N, A409R, A409D, A424E, A424V, P429S, A444T, I462T, G485V, W505S, W505R, A550D, F567S, A574E, A574V, P589T, G593D, M609I, G629S, A636E, G685R, G699Q, A713P, A716P, A727D
*pncA*	A146T, A171E, A171T, G162D, L159R, L182S, S179R, T142K, T153N, T168N
*gyrA*	A74S, G88A
*gyrB*	G509A, N538K, A543T, A543V

## Discussion

The present study proposes an AI/ML method to classify resistant and susceptible mutations in TB and predict novel resistance conferring mutations. The impact of the reported mutations was captured in the form of changes in the amino acid residues, and the consequent change in properties vis-a-vis wild-type and mutant proteins and represented as features used to train the models. The classification model was generated for each gene and predictions were made for SNVs linked with each gene for each drug. Four ML algorithms, NB, kNN, SVM, and ANN were used to generate learned model systems for genes associated with the first-line TB drugs rifampicin (*rpoB*), isoniazid (*katG* and *inhA*), pyrazinamide (*pncA*) and fluoroquinolones (*gyrA* and *gyrB*). The models were highly precise with average accuracies of 88.86%, 85.22%, 88.0%, 87.30%, 78.88%, and 86.88% for *rpoB*, *inhA*, *katG*, *pncA*, *gyrA*, and *gyrB*, respectively. Additionally, various feature selection algorithms were used to identify a subset of features having substantial involvement in the prediction task. We observed that ΔΔG ranked the highest among the ten features in classification for all genes except *gyrB*. This clearly indicated the importance of ΔΔG in all the classification models. The residue types also had a high correlation, demonstrating that the type of mutant residues significantly influenced the stability of protein. Hydrophobicity and polarity also played an important role in most of the prediction models, which is in line with the concept that the increased polarity and hydrophobic interactions contribute substantially to thermodynamic stability. The mutations predicted to resistance conferring were also analyzed for their impact on the conformation of the proteins upon binding with drugs, isoniazid, pyrazinamide, and fluoroquinolones, ofloxacin, moxifloxacin and ciprofloxacin. The interaction patterns observed in case of drug bound wild type and mutant proteins clearly indicated the destabilizing effect of mutations to a great extent in catalase peroxidase (*katG*) and pyrazinamidase (*pncA*) and moderately low in gyrase A (*gyrA*) and gyrase B (*gyrB*).

Conclusively, in the present work we have utilized the already existing information to train the computational models to predict the actual resistance-conferring mutations from the huge variation data resulting from high-throughput sequencing methods, genotyping techniques, and next-generation sequencing techniques. The models generated in the present study will predict any already existing mutation, be it rare or frequently occurring, as well as the novel mutations not encountered before. For each variation that is applied to the generated model, the model will predict it as resistance causing or susceptible using its previous knowledge, which is the various properties of the amino acids used in the present study. Thus, we believe that the AI and ML models generated in the present study will efficiently predict *M.tb* drug resistance and identify novel drug-associated mutations.

## Methods

### Dataset preparation

A list of nonsynonymous single nucleotide variations was obtained from the TBDReaMDB (Tuberculosis Drug Resistance Mutation Database)^34^ and GMTV (Genome-wide Mycobacterium tuberculosis Variation) Database Project^35^. TBDReaMDB is a broad spectrum database providing mutations associated with drug resistance in TB and their frequency of occurrence. The GMTV database is another wide-ranging database that contains data obtained from different sources related to *M.tb*, which lists the genetic markers associated with TB drug resistance profiles as well as clinical outcomes. The data were preprocessed, during which the mutations resulting in stop codon and self-mutated residues were discarded. The residues which were not present in the crystal structure of protein and the duplicates were also removed.

### Descriptors and labelling

The descriptor set used for the generation of the models included sequence and structure based features. The six physicochemical properties of the wild-type (wt) and mutant amino acid (AA) residues representing sequence based features included molecular weight, van der Waals volume, charge, isoelectric point, hydrophobicity scale, and residue type. The difference between the AA properties of the mutant and wild types were calculated and the resulting values were used as descriptors for the generation of the models. The normalized values for the amino acid properties were obtained from Gromiha^36^. The residue type for the mutant and wild type AAs was represented by four binary features (0, 1, 2, and 3) specifying whether the residues are charged (Asp, Lys, Glu, Arg), polar (Ser, Thr, Asn, Gln), aromatic (Phe, His, Trp, Tyr), or hydrophobic (Ala, Gly, Cys, Ile, Met, Leu, Val, Pro,), respectively.

The structural descriptors included the accessible surface area to determine the accessibility of the residue to the surface, the secondary structure of the residue at the mutation site indicating whether the mutation took place in helix, sheet, coil, or turn, and the free energy changes (ΔΔG) due to the mutation. The secondary structural features were represented as helix = 1, sheet = 2, coil = 3 and turn = 4. The following values for the accessible surface area were used: Ala-110.2; Arg-229.0; Asn-146.4; Asp-144.1; Cys-140.4; Gly-78.7; Gln-178.6; Glu-174.7; His-181.9; Ile-185.0; Leu-183.1; Lys-205.7; Met-200.1; Pro-141.9; Phe-200.7; Ser-117.2; Thr-138.7; Trp-240.5; Tyr-213.7; and Val-153.7 (the units are in Å2)^37,38^. The values for accessible surface area were normalized to the same range [0, 1] as the other features using the following equation

$$\frac{{x}-\,{\min }}{{\max }\,-\,{\min }}$$

where *x* is the value before denormalization, and *max* and *min* are the maximum and minimum values, respectively.

The normalized ΔΔG values for the residues were retrieved from Gromiha^36^. The mutations were classified as susceptible or resistant as a function of ΔΔG. A positive energy change upon mutation indicates the stability of the protein increased, whereas negative energy indicates a decrease in the stability of the protein structure. For classification purposes, mutations associated with a positive change in energy and thus stability were labeled as positive or susceptible mutations, while the others were labeled as negative or resistant mutations. Table 8 lists the type of descriptors used for the generation of the machine learning models.

**Table 8:** The types of descriptors used for the generation of machine learning models.

Sequence properties	Structural properties
Molecular weight; Polarity; Hydrophobicity; van der Waals volume; Residue type; Isoelectric point	Solvent accessible surface area; Secondary structure where the mutation is located in experimental structure; ΔΔG

### Machine learning algorithms

Different ML algorithms have different advantages. With this in mind, the following four supervised algorithms were used for prediction purposes: naïve Bayes (NB), k nearest neighbor (kNN), artificial neural network (ANN) and a support vector machine (SVM) based sequential minimization optimization (SMO) algorithm. The java based program Weka (Waikato Environment for Knowledge Analysis)^39^, a suite of ML algorithms for model building, was used.

The naïve Bayes (NB) classifier is a Bayes theorem based simple probabilistic classifier. The classifier assumes that each feature contributes independently toward classification and the value of every feature is independent of the value of any other feature. The NB classifier is fast, easy to build, and useful for the classification of large datasets. The classifier requires only a small quantity of data for training purposes and has worked well in many classification tasks out performing other algorithms such as random forest^40^.

K nearest neighbor (kNN) is the most basic ML algorithm and is frequently applied in data mining and pattern recognition. It has been successfully used for both classification and regression^41^. The algorithm chooses the k number of the closest objects from the feature space and calculates mainly the Euclidean distance between one object and its k nearest neighbors in training data. Further, it predicts the output class from the majority vote of those k nearest neighbors. KNN takes less calculation time and is easy to interpret as there is only a single parameter that needs to be tuned.

Sequential minimization optimization (SMO) is a fast new SVM algorithm, which is simple in concept and implementation with better scaling properties than the standard SVM algorithm. The SMO algorithm solves the large quadratic programming (QP) algorithm by breaking it into a sequence of smaller QP problems, which reduces computation time and enables SMO to handle large training sets instead of using numerical QP optimization steps as in the case of SVM^42^. The SVM algorithm in its simplest linear form uses a hyperplane that separates the positive from negative examples in a class by maximizing the margins. The margin is defined by calculating the distance between a hyperplane and the closest positive and negative example. In the case of non-linear classification, the algorithm uses the kernel function to transform the feature space and performs classification by projecting the inputs to high-dimensional feature spaces^43^.

Artificial neural network (ANN) is a computational model that attempts to mimic the structural and functional characteristics of biological neural networks^44,45^. It is a collection of nodes known as artificial neurons and the connection between nodes are the edges. Each artificial neuron and edges are associated with certain weights. An artificial neuron receives an input, activates and processes it using certain functions and then transfers it to the next neuron. The weights and functions that activate the neuron are modified by learning algorithms, which modify the parameters of the neural network to produce the desired output. A multilayer perceptron model was used, which is an implementation of the ANN algorithm in Weka.

### Predictive modelling

Two groups of features, sequence and structural, were used for model building. Default parameters were used to generate the models using NB, kNN, and ANN learning algorithms except in the case of SVM, in which a radial basis function kernel was used. Prior to model building the data were divided using an in-house Perl script with 80% for a training set and 20% for a testing set.

The internal validation of the models was performed using 10-fold cross validation. The training data were divided into 10-folds, of which 9 folds were used for training purposes while the remaining fold was used for evaluation of the model. This process was repeated until all the folds were used as test sets at least once. The performance of the models was further measured using the blind test set containing 20% of the data, which was not part of the training set used for generating the models.

### Statistical evaluation

The predictive performance of the classification models on the testing data was evaluated using accuracy and a receiver operating characteristic (ROC) curve from which area under curve (AUC) was also calculated. A ROC curve is a graphical plot created using the true positive rate and false positive rate, which demonstrates the predictive ability of the classifier models^46^.

### Descriptor selection

To identify the features having a significant role in the classification of resistant and susceptible mutations, feature selection was carried out using eight feature selection algorithms available in Weka. The eight feature selection techniques include symmetrical uncertainty based attribute evaluation, relief attribute evaluation, gain ratio and info gain algorithms, oneR classifier based algorithm, correlation based feature selection (CFS) algorithm, and classifier based attribute evaluation. The symmetrical uncertainty based attribute evaluation method calculates the significance of a feature by quantifying symmetrical uncertainty with respect to the prediction class. It selects the features in accordance with the value of an individual feature in the feature subset. The relief attribute evaluation method includes a sampling of instances over and over again until the value of the given attribute is same as the neighboring instances. Gain ratio and info gain attribute selection algorithms measure gain ratio and the information gain of the particular attribute with respect to the class, respectively^47^. The oneR algorithm selects features using a simple oneR algorithm that generates one rule for each predicting feature, then chooses the rule with the lowest total error as its ‘one rule’^48^. CFS evaluates the subsets of features based on the theory that a good feature subgroup contains descriptors with a high correlation to the class, however uncorrelated with each other.

### Molecular docking of wild type and predicted resistance causing mutant proteins with drugs

The X-ray crystal structures for wild type proteins *katG* (PDB ID: 1SJ2), *pncA* (PDB ID: 3PL1), *gyrA* (PDB ID: 4G3N (N-terminal) and 5BS8 (C-terminal)) and *gyrB* (PDB ID: 5BS8) were obtained from PDB^49^. The mutant models for the resistance causing mutations were generated using Schrodinger software^50^. The wild type and mutant models were then subjected to MD simulations to study the behavior of protein in the presence of external salts and solvents. The wild type and mutant proteins were preprocessed using Schrodinger’s Protein Preparation Wizard^51^, during which bond orders were corrected and hydrogen and disulfide bonds were added. The proteins were optimized at pH 7 using Propka^52^. The ligands used in the present study included drugs, isoniazid (PubChem CID: 3767), pyrazinamide (PubChem CID: 1046) and fluoroquinolones, ciprofloxacin (PubChem CID: 2764), moxifloxacin (PubChem CID: 152946) and ofloxacin (PubChem CID: 4583). LigPrep module was used for ligand preparation which generated energy minimized ligands using OPLS3 force field, possible tautomers and ionization states were created and the mistakes in the ligands were removed. The grid was created using Receptor Grid Generation module around the already predicted drug binding pocket and extra precision algorithm of Glide module was used to dock the ligands in the active site of the receptor. The docked protein-ligand complexes having lowest binding free energy values were taken for further investigation.

### Molecular dynamics simulations

In order to delineate the influence of predicted resistance causing mutations on the protein structure, the docked conformations were subjected to MD simulations conducted using GROMACS^53^ software, for which a GROMOS54A7 force field was used. After the initial preparation, the models were solvated with a simple point charge (SPC) water model and neutralized with the addition of Na^+^ and Cl^−^ ions to maintain the neutrality of the system. The solvated systems were then subjected to energy minimization using the steepest descent method, after which equilibration runs were performed in two consecutive steps, NVT (number of particles, volume and temperature) equilibration and NPT (number of particles, pressure and temperature) equilibration. Further, 10 ns MD simulation runs were carried out to obtain stable structures and time versus RMSD (root-mean square deviation) plots were generated. Rg, SASA and hydrogen bond (H-bond) were analyzed for wild type and mutant protein-drug complexes. The interaction images were generated using PyMol and UCSF Chimera.

## Supplementary information

Supplementary information.

Dataset 1.
