# Author Correction: Artificial Intelligence and Machine learning based prediction of resistant and susceptible mutations in *Mycobacterium tuberculosis*

**DOI:** 10.1038/s41598-020-71840-y

**Published:** 2020-09-01

**Authors:** Salma Jamal, Mohd. Khubaib, Rishabh Gangwar, Sonam Grover, Abhinav Grover, Seyed E. Hasnain

**Affiliations:** 1 Jamia Hamdard Institute of Molecular Medicine, Jamia Hamdard, Hamdard Nagar, New Delhi, 110062 India; 2 School of Biotechnology, Jawaharlal Nehru University, New Mehrauli Road, New Delhi, 110 067 India; 3 Dr. Reddy’s Institute of Life Sciences, University of Hyderabad Campus, Professor C.R. Rao Road, Hyderabad, 500046 India

Correction to: *Scientific Reports* 10.1038/s41598-020-62368-2, published online 26 March 2020

In this Article, Figure 7 is a duplication of Figure 10. The correct Figure 7 appears below.

**Figure 7:**
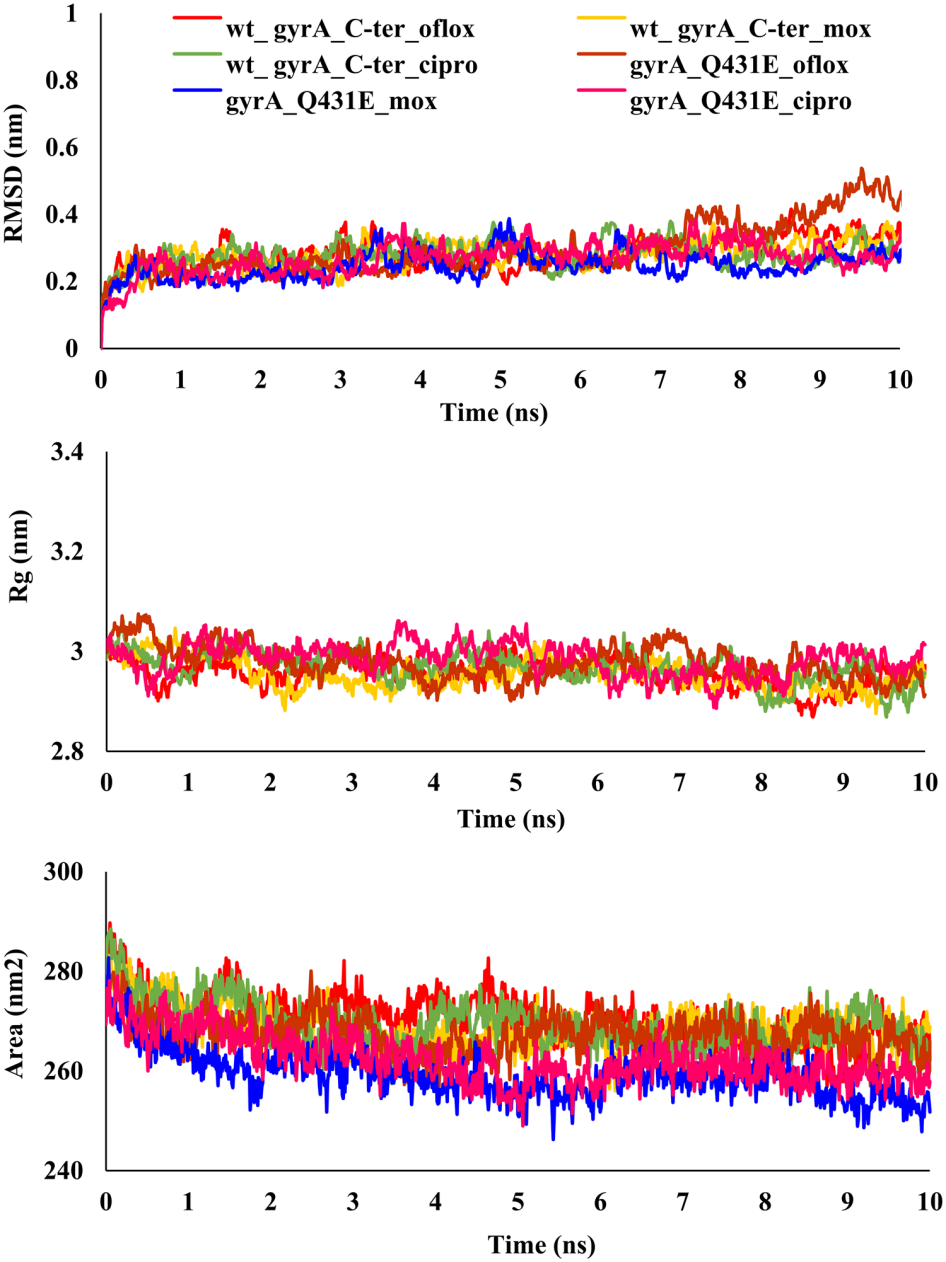
RMSD, Rg and SASA plot for* gyrA* gene, C-terminal protein. For Q431E mutant, the RMSD and Rg were slightly higher than wild type, however SASA was less for mutant protein.

