# Supplementary material for: Artificial Intelligence and Machine learning based prediction of resistant and susceptible mutations in Mycobacterium tuberculosis
Source: Sci Rep. 2020 Mar 26;10:5487. doi: 10.1038/s41598-020-62368-2 (PMC7099008; doi:10.1038/s41598-020-62368-2)
Supplement: Supplementary file 1 — Supplementary information. [file 41598_2020_62368_MOESM1_ESM.pdf]

Artificial Intelligence and Machine learning based prediction of resistant and susceptible mutations in *Mycobacterium tuberculosis*

Salma Jamal [1], Mohd. Khubaib [1], Rishabh Gangwar [1], Sonam Grover [1], Abhinav Grover [2] and Seyed E. Hasnain [1,3,*]

[1] Jamia Hamdard Institute of Molecular Medicine, Jamia Hamdard, Hamdard Nagar, New Delhi 110062; [2] School of Biotechnology, Jawaharlal Nehru University, New Mehrauli Road, New Delhi 110 067; [3] Dr Reddy's Institute of Life Sciences, University of Hyderabad Campus, Professor C.R. Rao Road, Hyderabad 500046

***To whom correspondence should be addressed**

Seyed E. Hasnain

Tel: (+91) 11-2605-9662 / 5216, 5213 (Office)

Email: vc@jamiahamdard.ac.in, seyedhasnain@gmail.com

Supplementary information

Supplementary table S1-S6. Final datasets comprising of variations and the descriptors used for model generation in the present study.

Supplementary table S7-S12. Ranking of the features selected by various feature selection techniques for all the genes, *rpoB*, *pncA*, *inhA*, *katG*, *gyrA* and *gyrB*.

Supplementary dataset 1. The AI/ML models generated in the present study.

Table S1. Descriptors_gyrA										
WT/Mutant	Molecular weight	Volume	Polarity	Isoelectric point	Hydrophobicity	Residue type(WT)	Residue type(Mt)	Secondary structure	Normalize ASA	ΔΔG (Mt-WT)
Phe64Leu	-0.27	-0.3	-0.01	0.06	-0.03	2	3	4	0.81	-0.78
His70Arg	0.15	0.1	0.01	0.4	-0.54	2	0	3	0.69	-0.44
Gly177Arg	0.77	0.7	1	0.6	-0.77	3	0	3	0	-0.28
His368Gln	-0.07	-0.1	-0.92	-0.24	-0.11	2	1	1	0.69	-0.27
Met438Ile	-0.14	0	-0.03	0.04	0.19	3	3	3	0.81	-0.21
Gln238Arg	0.22	0.2	0.93	0.64	-0.43	1	0	1	0.66	-0.17
Gly239Ser	0.23	0.2	0.03	-0.04	-0.17	3	1	1	0	-0.17
Gly247Ser	0.23	0.2	0.03	-0.04	-0.17	3	1	3	0	-0.17
Ala74Ser	0.12	0.1	0.03	-0.04	-0.21	3	1	1	0.21	-0.13
Ala463Ser	0.12	0.1	0.03	-0.04	-0.21	3	1	1	0.21	-0.13
Ala547Ser	0.12	0.1	0.03	-0.04	-0.21	3	1	3	0.21	-0.13
Pro472Ser	-0.08	-0.1	0	-0.08	-0.08	3	1	1	0.42	-0.12
Asp89Asn	-0.01	0	-0.89	0.33	-0.43	0	1	1	0.44	-0.08
Asp94Asn	-0.01	0	-0.89	0.33	-0.43	0	1	1	0.44	-0.08
Ala324Thr	0.23	0.2	0.03	-0.04	-0.18	3	1	1	0.21	-0.08
Ala445Thr	0.23	0.2	0.03	-0.04	-0.18	3	1	1	0.21	-0.08
Ile92Thr	-0.09	-0.1	0.03	-0.05	-0.37	3	1	1	0.71	-0.06
Pro108Gln	0.24	0.2	0.04	-0.08	-0.25	3	1	4	0.42	-0.06
Pro123Gln	0.24	0.2	0.04	-0.08	-0.25	3	1	3	0.42	-0.06
Gly668Asp	0.45	0.4	0.96	-0.4	0.11	3	0	4	0	-0.06
Gly694Asp	0.45	0.4	0.96	-0.4	0.11	3	0	4	0	-0.06
Thr135Ser	-0.11	-0.1	0	0	-0.03	1	1	3	0.4	-0.05
Gly63Ala	0.11	0.1	0	0	0.04	3	3	4	0	-0.04
Gly88Ala	0.11	0.1	0	0	0.04	3	3	3	0	-0.04
Gly744Ala	0.11	0.1	0	0	0.04	3	3	4	0	-0.04
Glu21Gln	-0.01	0	-0.89	0.05	-0.03	0	1	1	0.64	-0.03
Asn715Ser	-0.21	-0.2	-0.04	0.03	0.15	1	1	2	0.45	-0.03
Pro17Leu	0.12	0.1	-0.03	-0.04	0.24	3	3	3	0.42	-0.01
Ala90Val	0.22	0.2	0	0	0.11	3	3	1	0.21	-0.01
Ala384Val	0.22	0.2	0	0	0.11	3	3	1	0.21	-0.01
Val624Leu	0.1	0.1	0	0	0	3	3	2	0.5	-0.01
Asp94Val	-0.12	-0.1	-0.96	0.4	0.04	0	3	1	0.44	0.01
Leu96Pro	-0.12	-0.1	0.03	0.04	-0.24	3	3	1	0.69	0.01
Leu109Val	-0.1	-0.1	0	0	0	3	3	3	0.69	0.01
Pro124Ala	-0.2	-0.2	-0.03	-0.04	0.13	3	3	3	0.42	0.01
Leu296Pro	-0.12	-0.1	0.03	0.04	-0.24	3	3	4	0.69	0.01
Ile462Val	-0.1	-0.1	0	-0.01	-0.08	3	3	1	0.71	0.01
Asp94Ala	-0.34	-0.3	-0.96	0.4	-0.07	0	3	1	0.44	0.02
Glu386Val	-0.23	-0.2	-0.96	0.09	0.46	0	3	1	0.64	0.03
Gln431Glu	0.01	0	0.89	-0.05	0.03	1	0	1	0.66	0.03
Gln613Glu	0.01	0	0.89	-0.05	0.03	1	0	2	0.66	0.03
Ala90Gly	-0.11	-0.1	0	0	-0.04	3	3	1	0.21	0.04
Ser95Thr	0.11	0.1	0	0	0.03	1	1	1	0.26	0.05
Asp67Gly	-0.45	-0.4	-0.96	0.4	-0.11	0	3	4	0.44	0.06
Asp94Gly	-0.45	-0.4	-0.96	0.4	-0.11	0	3	1	0.44	0.06

Lys224Glu	0.01	0	0.01	-0.56	0.2	0	0	3	0.84	0.06
Asp359Gly	-0.45	-0.4	-0.96	0.4	-0.11	0	3	1	0.44	0.06
Thr80Ala	-0.23	-0.2	-0.03	0.04	0.18	1	3	1	0.4	0.08
Thr355Ala	-0.23	-0.2	-0.03	0.04	0.18	1	3	2	0.4	0.08
Ser684Leu	0.2	0.2	-0.03	0.04	0.32	1	3	2	0.26	0.11
Ser91Pro	0.08	0.1	0	0.08	0.08	1	3	1	0.26	0.12
Ser178Pro	0.08	0.1	0	0.08	0.08	1	3	2	0.26	0.12
Ser237Ala	-0.12	-0.1	-0.03	0.04	0.21	1	3	1	0.26	0.13
Ser250Ala	-0.12	-0.1	-0.03	0.04	0.21	1	3	2	0.26	0.13
Ser27Gly	-0.23	-0.2	-0.03	0.04	0.17	1	3	1	0.26	0.17
Arg309Gln	-0.22	-0.2	-0.93	-0.64	0.43	0	1	4	1	0.17
Arg578Gln	-0.22	-0.2	-0.93	-0.64	0.43	0	1	2	1	0.17
Pro102His	0.31	0.3	0.96	0.16	-0.14	3	2	4	0.42	0.21
Pro167His	0.31	0.3	0.96	0.16	-0.14	3	2	3	0.42	0.21
Leu711Met	0.14	0	0.03	-0.03	-0.11	3	3	2	0.69	0.21
Asp94His	0.17	0.2	0.03	0.6	-0.34	0	2	1	0.44	0.22
Arg252Leu	-0.34	-0.3	-1	-0.6	0.92	0	3	2	1	0.22
Arg292Leu	-0.34	-0.3	-1	-0.6	0.92	0	3	1	1	0.22
Arg376Leu	-0.34	-0.3	-1	-0.6	0.92	0	3	1	1	0.22
Arg392Leu	-0.34	-0.3	-1	-0.6	0.92	0	3	1	1	0.22
Arg292Gly	-0.77	-0.7	-1	-0.6	0.77	0	3	1	1	0.28
Arg382Gly	-0.77	-0.7	-1	-0.6	0.77	0	3	1	1	0.28
Gly88Cys	0.36	0.2	0.03	-0.11	-0.05	3	3	3	0	0.34
Asp94Tyr	0.37	0.4	-0.93	0.36	-0.17	0	2	1	0.44	0.34
Asp211Tyr	0.37	0.4	-0.93	0.36	-0.17	0	2	3	0.44	0.34
Arg495His	-0.15	-0.1	-0.01	-0.4	0.54	0	2	3	1	0.44
Asp94Phe	0.25	0.3	-0.95	0.34	0.07	0	2	1	0.44	0.78

Table S2. Descriptors_gyrB										
WT/Mutant	Molecular weight	Volume	Polarity	Isoelectric point	Hydrophobicity	Residue type(WT)	Residue type(Mt)	Secondary structure	Normalize ASA	ΔΔG (Mt-WT)
Trp174Leu	-0.57	-0.6	-0.04	0.01	0.07	2	3	2	1	-0.65
Met652Thr	-0.23	-0.1	0	-0.01	-0.18	3	1	1	0.75	-0.27
Met58Ile	-0.14	0	-0.03	0.04	0.19	3	3	1	0.75	-0.21
Met291Ile	-0.14	0	-0.03	0.04	0.19	3	3	2	0.75	-0.21
His394Pro	-0.31	-0.3	-0.96	-0.16	0.14	2	3	1	0.64	-0.21
Ala78Ser	0.12	0.1	0.03	-0.04	-0.21	3	1	2	0.19	-0.13
Ala162Ser	0.12	0.1	0.03	-0.04	-0.21	3	1	2	0.19	-0.13
Ala403Ser	0.12	0.1	0.03	-0.04	-0.21	3	1	1	0.19	-0.13
Asp461Asn	-0.01	0	-0.89	0.33	-0.43	0	1	1	0.4	-0.08
Asp472Asn	-0.01	0	-0.89	0.33	-0.43	0	1	4	0.4	-0.08
Ala504Thr	0.23	0.2	0.03	-0.04	-0.18	3	1	1	0.19	-0.08
Glu71Lys	-0.01	0	-0.01	0.56	-0.2	0	0	4	0.59	-0.06
Gly73Val	0.33	0.3	0	0	0.15	3	3	4	0	-0.05
Gly520Ala	0.11	0.1	0	0	0.04	3	3	1	0	-0.04
Asn499Ser	-0.21	-0.2	-0.04	0.03	0.15	1	1	3	0.42	-0.03
Asp97Glu	0.11	0.1	0	0.31	-0.42	0	0	1	0.4	-0.02
Asp259Glu	0.11	0.1	0	0.31	-0.42	0	0	1	0.4	-0.02
Asp639Glu	0.11	0.1	0	0.31	-0.42	0	0	4	0.4	-0.02
Ala644Asp	0.34	0.3	0.96	-0.4	0.07	3	0	1	0.19	-0.02
Ala62Val	0.22	0.2	0	0	0.11	3	3	3	0.19	-0.01
Pro94Leu	0.12	0.1	-0.03	-0.04	0.24	3	3	2	0.39	-0.01
Pro177Leu	0.12	0.1	-0.03	-0.04	0.24	3	3	4	0.39	-0.01
Ala242Pro	0.2	0.2	0.03	0.04	-0.13	3	3	3	0.19	-0.01
Val301Leu	0.1	0.1	0	0	0	3	3	2	0.46	-0.01
Ala398Val	0.22	0.2	0	0	0.11	3	3	1	0.19	-0.01
Ala504Val	0.22	0.2	0	0	0.11	3	3	1	0.19	-0.01
Pro554Leu	0.12	0.1	-0.03	-0.04	0.24	3	3	1	0.39	-0.01
Ala593Val	0.22	0.2	0	0	0.11	3	3	1	0.19	-0.01
Lys159Asn	-0.11	-0.1	-0.88	-0.54	0.19	0	1	2	0.78	0
Lys268Asn	-0.11	-0.1	-0.88	-0.54	0.19	0	1	4	0.78	0
Lys441Asn	-0.11	-0.1	-0.88	-0.54	0.19	0	1	4	0.78	0
Lys596Asn	-0.11	-0.1	-0.88	-0.54	0.19	0	1	1	0.78	0
Lys603Asn	-0.11	-0.1	-0.88	-0.54	0.19	0	1	4	0.78	0
Asp472Ala	-0.34	-0.3	-0.96	0.4	-0.07	0	3	4	0.4	0.02
Asn499Thr	-0.1	-0.1	-0.04	0.03	0.18	1	1	3	0.42	0.02
Glu501Asp	-0.11	-0.1	0	-0.31	0.42	0	0	1	0.59	0.02
Thr500Ile	0.09	0.1	-0.03	0.05	0.37	1	3	1	0.37	0.06
Thr511Pro	-0.03	0	0	0.08	0.05	1	3	3	0.37	0.07
Thr500Ala	-0.23	-0.2	-0.03	0.04	0.18	1	3	1	0.37	0.08
Ser576Gly	-0.23	-0.2	-0.03	0.04	0.17	1	3	4	0.24	0.17
Asp472His	0.17	0.2	0.03	0.6	-0.34	0	2	4	0.4	0.22

Arg40Pro	-0.46	-0.4	-0.97	-0.56	0.68	0	3	1	0.93	0.23
Asn499Tyr	0.38	0.4	-0.04	0.03	0.26	1	2	3	0.42	0.42
Arg421His	-0.15	-0.1	-0.01	-0.4	0.54	0	2	1	0.93	0.44
Arg446Cys	-0.41	-0.5	-0.97	-0.71	0.72	0	3	3	0.93	0.62
Val670Phe	0.37	0.4	0.01	-0.06	0.03	3	2	4	0.46	0.77
Arg609Trp	0.23	0.3	-0.96	-0.61	0.85	0	2	2	0.93	0.87
Ser447Phe	0.47	0.5	-0.02	-0.02	0.35	1	2	3	0.24	0.89
Ser649Phe	0.47	0.5	-0.02	-0.02	0.35	1	2	1	0.24	0.89

Table S3. Descriptors_inhA										
WT/Mutant	Molecular weight	Volume	Polarity	Isoelectric point	Hydrophobicity	Residue type(WT)	Residue type(Mt)	Secondary structure	Normalize ASA	ΔΔG (Mt-WT)
His265Asp	-0.17	-0.2	-0.03	-0.6	0.34	2	0	1	0.69	-0.22
Gln35Arg	0.22	0.2	0.93	0.64	-0.43	1	0	2	0.66	-0.17
Gly85Ser	0.23	0.2	0.03	-0.04	-0.17	3	1	4	0	-0.17
Gln216Arg	0.22	0.2	0.93	0.64	-0.43	1	0	1	0.66	-0.17
Ile16Thr	-0.09	-0.1	0.03	-0.05	-0.37	3	1	3	0.71	-0.06
Ile21Thr	-0.09	-0.1	0.03	-0.05	-0.37	3	1	1	0.71	-0.06
Ile47Thr	-0.09	-0.1	0.03	-0.05	-0.37	3	1	1	0.71	-0.06
Ile95Thr	-0.09	-0.1	0.03	-0.05	-0.37	3	1	3	0.71	-0.06
Ile194Thr	-0.09	-0.1	0.03	-0.05	-0.37	3	1	4	0.71	-0.06
Ala124Glu	0.45	0.4	0.96	-0.09	-0.35	3	0	1	0.21	-0.04
Lys8Asn	-0.11	-0.1	-0.88	-0.54	0.19	0	1	2	0.84	0
Lys57Asn	-0.11	-0.1	-0.88	-0.54	0.19	0	1	3	0.84	0
Leu61Ile	0	0	0	0.01	0.08	3	3	2	0.69	0
Phe97Phe	0	0	0	0	0	2	2	3	0.81	0
Gly255Gly	0	0	0	0	0	3	3	4	0	0
Ile21Val	-0.1	-0.1	0	-0.01	-0.08	3	3	1	0.71	0.01
Val78Ala	-0.22	-0.2	0	0	-0.11	3	3	1	0.5	0.01
Ile95Pro	-0.12	-0.1	0.03	0.03	-0.32	3	3	3	0.71	0.01
Leu61Val	-0.1	-0.1	0	0	0	3	3	2	0.69	0.01
Ile228Val	-0.1	-0.1	0	-0.01	-0.08	3	3	4	0.71	0.01
Pro251Ala	-0.2	-0.2	-0.03	-0.04	0.13	3	3	4	0.42	0.01
Lys118Glu	0.01	0	0.01	-0.56	0.2	0	0	1	0.84	0.06
Asn231Asp	0.01	0	0.89	-0.33	0.43	1	0	4	0.45	0.08
Ser94Leu	0.2	0.2	-0.03	0.04	0.32	1	3	3	0.26	0.11
Ser94Ala	-0.12	-0.1	-0.03	0.04	0.21	1	3	3	0.26	0.13
Val65Met	0.24	0.1	0.03	-0.03	-0.11	3	3	4	0.5	0.2
Asp234Tyr	0.37	0.4	-0.93	0.36	-0.17	0	2	4	0.44	0.34

Table S4. Descriptors_katG										
WT/Mutant	Molecular weight	Volume	Polarity	Isoelectric point	Hydrophobicity	Residue type(WT)	Residue type(Mt)	Secondary structure	Normalize ASA	ΔΔG (Mt-WT)
Arg104Trp	0.23	0.3	-0.96	-0.61	0.85	0	2	1	0.93	0.87
Leu141Phe	0.27	0.3	0.01	-0.06	0.03	3	2	1	0.65	0.78
Asp311Phe	0.25	0.3	-0.95	0.34	0.07	0	2	1	0.4	0.78
Leu449Phe	0.27	0.3	0.01	-0.06	0.03	3	2	3	0.65	0.78
Leu634Phe	0.27	0.3	0.01	-0.06	0.03	3	2	1	0.65	0.78
Val469Phe	0.37	0.4	0.01	-0.06	0.03	3	2	1	0.46	0.77
Gly309Phe	0.7	0.7	0.01	-0.06	0.18	3	2	1	0	0.72
Arg249Cys	-0.41	-0.5	-0.97	-0.71	0.72	0	3	1	0.93	0.62
Arg515Cys	-0.41	-0.5	-0.97	-0.71	0.72	0	3	1	0.93	0.62
Ser331Cys	0.13	0	0	-0.07	0.12	1	3	1	0.24	0.51
Thr180Cys	0.02	-0.1	0	-0.07	0.09	1	3	3	0.37	0.46
Tyr229Phe	-0.12	-0.1	-0.02	-0.02	0.24	2	2	4	0.83	0.44
Tyr337Phe	-0.12	-0.1	-0.02	-0.02	0.24	2	2	1	0.83	0.44
Arg571His	-0.15	-0.1	-0.01	-0.4	0.54	0	2	3	0.93	0.44
Asp74Tyr	0.37	0.4	-0.93	0.36	-0.17	0	2	1	0.4	0.34
Gly96Cys	0.36	0.2	0.03	-0.11	-0.05	3	3	1	0	0.34
Gly121Cys	0.36	0.2	0.03	-0.11	-0.05	3	3	3	0	0.34
Gly125Cys	0.36	0.2	0.03	-0.11	-0.05	3	3	4	0	0.34
Gly299Cys	0.36	0.2	0.03	-0.11	-0.05	3	3	3	0	0.34
Gly309Cys	0.36	0.2	0.03	-0.11	-0.05	3	3	1	0	0.34
Asp311Tyr	0.37	0.4	-0.93	0.36	-0.17	0	2	1	0.4	0.34
Gly491Cys	0.36	0.2	0.03	-0.11	-0.05	3	3	3	0	0.34
Gly494Cys	0.36	0.2	0.03	-0.11	-0.05	3	3	2	0	0.34
Asn138His	0.18	0.2	0.92	0.27	0.09	1	2	1	0.42	0.3
Thr251Met	0.23	0.1	0	0.01	0.18	1	3	1	0.37	0.27
Gln502His	0.07	0.1	0.92	0.24	0.11	1	2	1	0.62	0.27
Arg128Pro	-0.46	-0.4	-0.97	-0.56	0.68	0	3	1	0.93	0.23
Arg104Leu	-0.34	-0.3	-1	-0.6	0.92	0	3	1	0.93	0.22
Asp357His	0.17	0.2	0.03	0.6	-0.34	0	2	4	0.4	0.22
Arg418Leu	-0.34	-0.3	-1	-0.6	0.92	0	3	1	0.93	0.22
Asp419His	0.17	0.2	0.03	0.6	-0.34	0	2	3	0.4	0.22
Arg463Leu	-0.34	-0.3	-1	-0.6	0.92	0	3	1	0.93	0.22
Arg496Leu	-0.34	-0.3	-1	-0.6	0.92	0	3	1	0.93	0.22
Leu141Met	0.14	0	0.03	-0.03	-0.11	3	3	1	0.65	0.21
Pro280His	0.31	0.3	0.96	0.16	-0.14	3	2	4	0.39	0.21
Leu587Met	0.14	0	0.03	-0.03	-0.11	3	3	1	0.65	0.21
Val586Met	0.24	0.1	0.03	-0.03	-0.11	3	3	1	0.46	0.2
Arg104Gln	-0.22	-0.2	-0.93	-0.64	0.43	0	1	1	0.93	0.17
Ser140Gly	-0.23	-0.2	-0.03	0.04	0.17	1	3	4	0.24	0.17
Ser315Gly	-0.23	-0.2	-0.03	0.04	0.17	1	3	4	0.24	0.17
Arg418Gln	-0.22	-0.2	-0.93	-0.64	0.43	0	1	1	0.93	0.17

Gly186His	0.62	0.6	0.99	0.2	-0.23	3	2	3	0	0.16
Ser140Ala	-0.12	-0.1	-0.03	0.04	0.21	1	3	4	0.24	0.13
Ser383Ala	-0.12	-0.1	-0.03	0.04	0.21	1	3	1	0.24	0.13
Ser700Pro	0.08	0.1	0	0.08	0.08	1	3	1	0.24	0.12
Ser160Leu	0.2	0.2	-0.03	0.04	0.32	1	3	3	0.24	0.11
Ser315Ile	0.2	0.2	-0.03	0.05	0.4	1	3	4	0.24	0.11
Ser315Leu	0.2	0.2	-0.03	0.04	0.32	1	3	4	0.24	0.11
Ser457Ile	0.2	0.2	-0.03	0.05	0.4	1	3	1	0.24	0.11
Arg484Ser	-0.54	-0.5	-0.97	-0.64	0.6	0	1	2	0.93	0.11
Ser527Leu	0.2	0.2	-0.03	0.04	0.32	1	3	1	0.24	0.11
Asn35Asp	0.01	0	0.89	-0.33	0.43	1	0	1	0.42	0.08
Asn138Asp	0.01	0	0.89	-0.33	0.43	1	0	1	0.42	0.08
Asn218Ile	-0.01	0	-0.07	0.08	0.55	1	3	4	0.42	0.08
Thr275Ala	-0.23	-0.2	-0.03	0.04	0.18	1	3	3	0.37	0.08
Thr394Ala	-0.23	-0.2	-0.03	0.04	0.18	1	3	1	0.37	0.08
Asn529Asp	0.01	0	0.89	-0.33	0.43	1	0	1	0.42	0.08
Thr85Pro	-0.03	0	0	0.08	0.05	1	3	1	0.37	0.07
Thr86Pro	-0.03	0	0	0.08	0.05	1	3	3	0.37	0.07
Thr112Pro	-0.03	0	0	0.08	0.05	1	3	4	0.37	0.07
Thr275Pro	-0.03	0	0	0.08	0.05	1	3	3	0.37	0.07
Thr308Pro	-0.03	0	0	0.08	0.05	1	3	3	0.37	0.07
Thr324Pro	-0.03	0	0	0.08	0.05	1	3	4	0.37	0.07
Asp72Gly	-0.45	-0.4	-0.96	0.4	-0.11	0	3	3	0.4	0.06
Asp74Gly	-0.45	-0.4	-0.96	0.4	-0.11	0	3	1	0.4	0.06
Asp94Gly	-0.45	-0.4	-0.96	0.4	-0.11	0	3	1	0.4	0.06
Gln127Pro	-0.24	-0.2	-0.04	0.08	0.25	1	3	1	0.62	0.06
Asp142Gly	-0.45	-0.4	-0.96	0.4	-0.11	0	3	1	0.4	0.06
Tyr155Cys	-0.46	-0.6	0	-0.07	0.01	2	3	1	0.83	0.06
Asp259Gly	-0.45	-0.4	-0.96	0.4	-0.11	0	3	1	0.4	0.06
Gln295Pro	-0.24	-0.2	-0.04	0.08	0.25	1	3	1	0.62	0.06
Asp311Gly	-0.45	-0.4	-0.96	0.4	-0.11	0	3	1	0.4	0.06
Tyr337Cys	-0.46	-0.6	0	-0.07	0.01	2	3	1	0.83	0.06
Thr380Ile	0.09	0.1	-0.03	0.05	0.37	1	3	1	0.37	0.06
Asp381Gly	-0.45	-0.4	-0.96	0.4	-0.11	0	3	1	0.4	0.06
Gln434Pro	-0.24	-0.2	-0.04	0.08	0.25	1	3	3	0.62	0.06
Gln525Pro	-0.24	-0.2	-0.04	0.08	0.25	1	3	1	0.62	0.06
Lys537Glu	0.01	0	0.01	-0.56	0.2	0	0	3	0.78	0.06
Gln717Pro	-0.24	-0.2	-0.04	0.08	0.25	1	3	1	0.62	0.06
Val68Gly	-0.33	-0.3	0	0	-0.15	3	3	1	0.46	0.05
Ser315Thr	0.11	0.1	0	0	0.03	1	1	4	0.24	0.05
Gln471Leu	-0.12	-0.1	-0.07	0.04	0.49	1	3	1	0.62	0.05
Ala551Gly	-0.11	-0.1	0	0	-0.04	3	3	1	0.19	0.04
Ser140Asn	0.21	0.2	0.04	-0.03	-0.15	1	1	4	0.24	0.03
Gln224Glu	0.01	0	0.89	-0.05	0.03	1	0	4	0.62	0.03
Ser315Asn	0.21	0.2	0.04	-0.03	-0.15	1	1	4	0.24	0.03
Asp94Ala	-0.34	-0.3	-0.96	0.4	-0.07	0	3	1	0.4	0.02
Asp117Ala	-0.34	-0.3	-0.96	0.4	-0.07	0	3	4	0.4	0.02

Asn138Thr	-0.1	-0.1	-0.04	0.03	0.18	1	1	1	0.42	0.02
Asp142Ala	-0.34	-0.3	-0.96	0.4	-0.07	0	3	1	0.4	0.02
Lys143Thr	-0.21	-0.2	-0.92	-0.51	0.37	0	1	1	0.78	0.02
Leu148Ala	-0.32	-0.3	0	0	-0.11	3	3	1	0.65	0.02
Asn236Thr	-0.1	-0.1	-0.04	0.03	0.18	1	1	1	0.42	0.02
Glu289Asp	-0.11	-0.1	0	-0.31	0.42	0	0	1	0.59	0.02
Lys345Thr	-0.21	-0.2	-0.92	-0.51	0.37	0	1	2	0.78	0.02
Asp509Ala	-0.34	-0.3	-0.96	0.4	-0.07	0	3	3	0.4	0.02
Asp695Ala	-0.34	-0.3	-0.96	0.4	-0.07	0	3	1	0.4	0.02
Asp735Ala	-0.34	-0.3	-0.96	0.4	-0.07	0	3	4	0.4	0.02
Asp215Val	-0.12	-0.1	-0.96	0.4	0.04	0	3	2	0.4	0.01
Val230Ala	-0.22	-0.2	0	0	-0.11	3	3	4	0.46	0.01
Ile335Val	-0.1	-0.1	0	-0.01	-0.08	3	3	1	0.66	0.01
Leu378Pro	-0.12	-0.1	0.03	0.04	-0.24	3	3	3	0.65	0.01
Leu514Pro	-0.12	-0.1	0.03	0.04	-0.24	3	3	1	0.65	0.01
Leu587Pro	-0.12	-0.1	0.03	0.04	-0.24	3	3	1	0.65	0.01
Leu619Pro	-0.12	-0.1	0.03	0.04	-0.24	3	3	3	0.65	0.01
Leu653Pro	-0.12	-0.1	0.03	0.04	-0.24	3	3	3	0.65	0.01
Val710Ala	-0.22	-0.2	0	0	-0.11	3	3	1	0.46	0.01
Leu205Ile	0	0	0	0.01	0.08	3	3	4	0.65	0
Asn218Lys	0.11	0.1	0.88	0.54	-0.19	1	0	4	0.42	0
Asn238Lys	0.11	0.1	0.88	0.54	-0.19	1	0	4	0.42	0
Ile317Leu	0	0	0	-0.01	-0.08	3	3	3	0.66	0
Lys414Asn	-0.11	-0.1	-0.88	-0.54	0.19	0	1	1	0.78	0
Lys488Asn	-0.11	-0.1	-0.88	-0.54	0.19	0	1	4	0.78	0
Asp580Asp	0	0	0	0	0	0	0	3	0.4	0
Leu587Ile	0	0	0	0.01	0.08	3	3	1	0.65	0
Ala66Pro	0.2	0.2	0.03	0.04	-0.13	3	3	1	0.19	-0.01
Ala106Val	0.22	0.2	0	0	0.11	3	3	1	0.19	-0.01
Ala109Val	0.22	0.2	0	0	0.11	3	3	1	0.19	-0.01
Ala110Val	0.22	0.2	0	0	0.11	3	3	1	0.19	-0.01
Ala122Val	0.22	0.2	0	0	0.11	3	3	4	0.19	-0.01
Ala139Pro	0.2	0.2	0.03	0.04	-0.13	3	3	4	0.19	-0.01
Ala172Val	0.22	0.2	0	0	0.11	3	3	1	0.19	-0.01
Ala281Val	0.22	0.2	0	0	0.11	3	3	4	0.19	-0.01
Ala291Pro	0.2	0.2	0.03	0.04	-0.13	3	3	3	0.19	-0.01
Ala379Val	0.22	0.2	0	0	0.11	3	3	1	0.19	-0.01
Val423Ile	0.1	0.1	0	0.01	0.08	3	3	1	0.46	-0.01
Ala424Val	0.22	0.2	0	0	0.11	3	3	1	0.19	-0.01
Val450Ile	0.1	0.1	0	0.01	0.08	3	3	3	0.46	-0.01
Ala479Val	0.22	0.2	0	0	0.11	3	3	1	0.19	-0.01
Ala574Val	0.22	0.2	0	0	0.11	3	3	3	0.19	-0.01
Pro603Leu	0.12	0.1	-0.03	-0.04	0.24	3	3	3	0.39	-0.01
Ala713Pro	0.2	0.2	0.03	0.04	-0.13	3	3	4	0.19	-0.01
Ala716Pro	0.2	0.2	0.03	0.04	-0.13	3	3	1	0.19	-0.01
Asp63Glu	0.11	0.1	0	0.31	-0.42	0	0	3	0.4	-0.02
Thr180Lys	0.21	0.2	0.92	0.51	-0.37	1	0	3	0.37	-0.02

Asp311Glu	0.11	0.1	0	0.31	-0.42	0	0	1	0.4	-0.02
Ala361Asp	0.34	0.3	0.96	-0.4	0.07	3	0	4	0.19	-0.02
Ala409Asp	0.34	0.3	0.96	-0.4	0.07	3	0	1	0.19	-0.02
Ala550Asp	0.34	0.3	0.96	-0.4	0.07	3	0	1	0.19	-0.02
Ala727Asp	0.34	0.3	0.96	-0.4	0.07	3	0	1	0.19	-0.02
Asn138Ser	-0.21	-0.2	-0.04	0.03	0.15	1	1	1	0.42	-0.03
Asn258Ser	-0.21	-0.2	-0.04	0.03	0.15	1	1	3	0.42	-0.03
Gly111Ala	0.11	0.1	0	0	0.04	3	3	4	0	-0.04
Gly169Ala	0.11	0.1	0	0	0.04	3	3	1	0	-0.04
Gly299Ala	0.11	0.1	0	0	0.04	3	3	3	0	-0.04
Gly305Ala	0.11	0.1	0	0	0.04	3	3	4	0	-0.04
Gly307Ala	0.11	0.1	0	0	0.04	3	3	4	0	-0.04
Ala424Glu	0.45	0.4	0.96	-0.09	-0.35	3	0	1	0.19	-0.04
Ala574Glu	0.45	0.4	0.96	-0.09	-0.35	3	0	3	0.19	-0.04
Ala636Glu	0.45	0.4	0.96	-0.09	-0.35	3	0	3	0.19	-0.04
Leu48Gln	0.12	0.1	0.07	-0.04	-0.49	3	1	4	0.65	-0.05
Gly121Val	0.33	0.3	0	0	0.15	3	3	3	0	-0.05
Gly186Val	0.33	0.3	0	0	0.15	3	3	3	0	-0.05
Thr271Ser	-0.11	-0.1	0	0	-0.03	1	1	4	0.37	-0.05
Thr275Ser	-0.11	-0.1	0	0	-0.03	1	1	3	0.37	-0.05
Gly285Val	0.33	0.3	0	0	0.15	3	3	2	0	-0.05
Gly297Val	0.33	0.3	0	0	0.15	3	3	4	0	-0.05
Gly485Val	0.33	0.3	0	0	0.15	3	3	4	0	-0.05
Thr690Ser	-0.11	-0.1	0	0	-0.03	1	1	2	0.37	-0.05
Glu195Lys	-0.01	0	-0.01	0.56	-0.2	0	0	3	0.59	-0.06
Gly279Asp	0.45	0.4	0.96	-0.4	0.11	3	0	3	0	-0.06
Gly285Asp	0.45	0.4	0.96	-0.4	0.11	3	0	2	0	-0.06
Gly309Asp	0.45	0.4	0.96	-0.4	0.11	3	0	1	0	-0.06
Gly316Asp	0.45	0.4	0.96	-0.4	0.11	3	0	4	0	-0.06
Glu318Lys	-0.01	0	-0.01	0.56	-0.2	0	0	3	0.59	-0.06
Ile335Thr	-0.09	-0.1	0.03	-0.05	-0.37	3	1	1	0.66	-0.06
Ile462Thr	-0.09	-0.1	0.03	-0.05	-0.37	3	1	1	0.66	-0.06
Glu523Lys	-0.01	0	-0.01	0.56	-0.2	0	0	1	0.59	-0.06
Gly593Asp	0.45	0.4	0.96	-0.4	0.11	3	0	1	0	-0.06
Glu607Lys	-0.01	0	-0.01	0.56	-0.2	0	0	1	0.59	-0.06
Gly644Asp	0.45	0.4	0.96	-0.4	0.11	3	0	4	0	-0.06
Pro241Thr	0.03	0	0	-0.08	-0.05	3	1	1	0.39	-0.07
Pro589Thr	0.03	0	0	-0.08	-0.05	3	1	4	0.39	-0.07
Ala60Thr	0.23	0.2	0.03	-0.04	-0.18	3	1	4	0.19	-0.08
Ala61Thr	0.23	0.2	0.03	-0.04	-0.18	3	1	4	0.19	-0.08
Ala65Thr	0.23	0.2	0.03	-0.04	-0.18	3	1	1	0.19	-0.08
Ile71Asn	0.01	0	0.07	-0.08	-0.55	3	1	3	0.66	-0.08
Asp72Lys	0.1	0.1	-0.01	0.87	-0.62	0	0	3	0.4	-0.08
Gly99Glu	0.56	0.5	0.96	-0.09	-0.31	3	0	1	0	-0.08
Ala109Thr	0.23	0.2	0.03	-0.04	-0.18	3	1	1	0.19	-0.08
Ala162Thr	0.23	0.2	0.03	-0.04	-0.18	3	1	1	0.19	-0.08
Ala172Thr	0.23	0.2	0.03	-0.04	-0.18	3	1	1	0.19	-0.08

Gly234Glu	0.56	0.5	0.96	-0.09	-0.31	3	0	4	0	-0.08
Ala264Thr	0.23	0.2	0.03	-0.04	-0.18	3	1	1	0.19	-0.08
Gly307Glu	0.56	0.5	0.96	-0.09	-0.31	3	0	4	0	-0.08
Ala350Thr	0.23	0.2	0.03	-0.04	-0.18	3	1	3	0.19	-0.08
Asp357Asn	-0.01	0	-0.89	0.33	-0.43	0	1	4	0.4	-0.08
Ile393Asn	0.01	0	0.07	-0.08	-0.55	3	1	1	0.66	-0.08
Ala444Thr	0.23	0.2	0.03	-0.04	-0.18	3	1	3	0.19	-0.08
Asp573Asn	-0.01	0	-0.89	0.33	-0.43	0	1	2	0.4	-0.08
Asp735Asn	-0.01	0	-0.89	0.33	-0.43	0	1	4	0.4	-0.08
Ser140Arg	0.54	0.5	0.97	0.64	-0.6	1	0	4	0.24	-0.11
Ser302Arg	0.54	0.5	0.97	0.64	-0.6	1	0	2	0.24	-0.11
Ser315Arg	0.54	0.5	0.97	0.64	-0.6	1	0	4	0.24	-0.11
Gly699Gln	0.55	0.5	0.07	-0.04	-0.34	3	1	1	0	-0.11
Tyr413His	-0.2	-0.2	0.96	0.24	-0.17	2	2	1	0.83	-0.12
Pro429Ser	-0.08	-0.1	0	-0.08	-0.08	3	1	4	0.39	-0.12
Ala243Ser	0.12	0.1	0.03	-0.04	-0.21	3	1	1	0.19	-0.13
Ala245Ser	0.12	0.1	0.03	-0.04	-0.21	3	1	1	0.19	-0.13
Ala350Ser	0.12	0.1	0.03	-0.04	-0.21	3	1	3	0.19	-0.13
Ala727Ser	0.12	0.1	0.03	-0.04	-0.21	3	1	1	0.19	-0.13
Thr262Arg	0.43	0.4	0.97	0.64	-0.63	1	0	1	0.37	-0.16
Gln88Arg	0.22	0.2	0.93	0.64	-0.43	1	0	2	0.62	-0.17
Gly120Ser	0.23	0.2	0.03	-0.04	-0.17	3	1	3	0	-0.17
Gly299Ser	0.23	0.2	0.03	-0.04	-0.17	3	1	3	0	-0.17
Gly309Ser	0.23	0.2	0.03	-0.04	-0.17	3	1	1	0	-0.17
Gly316Ser	0.23	0.2	0.03	-0.04	-0.17	3	1	4	0	-0.17
Gly629Ser	0.23	0.2	0.03	-0.04	-0.17	3	1	1	0	-0.17
Met242Val	-0.24	-0.1	-0.03	0.03	0.11	3	3	1	0.75	-0.2
Glu454Arg	0.21	0.2	0.04	0.69	-0.46	0	0	1	0.59	-0.2
Met84Ile	-0.14	0	-0.03	0.04	0.19	3	3	1	0.75	-0.21
Met176Ile	-0.14	0	-0.03	0.04	0.19	3	3	1	0.75	-0.21
Met257Ile	-0.14	0	-0.03	0.04	0.19	3	3	3	0.75	-0.21
His108Asp	-0.17	-0.2	-0.03	-0.6	0.34	2	0	1	0.64	-0.22
Leu148Arg	0.34	0.3	1	0.6	-0.92	3	0	1	0.65	-0.22
Leu336Arg	0.34	0.3	1	0.6	-0.92	3	0	1	0.65	-0.22
Leu384Arg	0.34	0.3	1	0.6	-0.92	3	0	1	0.65	-0.22
Leu437Arg	0.34	0.3	1	0.6	-0.92	3	0	1	0.65	-0.22
Ala409Arg	0.66	0.6	1	0.6	-0.81	3	0	1	0.19	-0.24
Trp321Cys	-0.64	-0.8	-0.01	-0.1	-0.13	2	3	3	1	-0.25
Trp328Cys	-0.64	-0.8	-0.01	-0.1	-0.13	2	3	3	1	-0.25
Trp728Cys	-0.64	-0.8	-0.01	-0.1	-0.13	2	3	1	1	-0.25
His108Gln	-0.07	-0.1	-0.92	-0.24	-0.11	2	1	1	0.64	-0.27
Met257Thr	-0.23	-0.1	0	-0.01	-0.18	3	1	3	0.75	-0.27
Gly234Arg	0.77	0.7	1	0.6	-0.77	3	0	4	0	-0.28
Gly307Arg	0.77	0.7	1	0.6	-0.77	3	0	4	0	-0.28
Gly685Arg	0.77	0.7	1	0.6	-0.77	3	0	4	0	-0.28
Phe129Cys	-0.34	-0.5	0.02	-0.05	-0.23	2	3	4	0.75	-0.38
Tyr155Ser	-0.59	-0.6	0	0	-0.11	2	1	1	0.83	-0.45

Tyr304Ser	-0.59	-0.6	0	0	-0.11	2	1	4	0.83	-0.45
Trp300Gly	-1	-1	-0.04	0.01	-0.08	2	3	3	1	-0.59
Trp328Gly	-1	-1	-0.04	0.01	-0.08	2	3	3	1	-0.59
Trp321Leu	-0.57	-0.6	-0.04	0.01	0.07	2	3	3	1	-0.65
Trp328Leu	-0.57	-0.6	-0.04	0.01	0.07	2	3	3	1	-0.65
Trp505Leu	-0.57	-0.6	-0.04	0.01	0.07	2	3	3	1	-0.65
Trp321Ser	-0.77	-0.8	-0.01	-0.03	-0.25	2	1	3	1	-0.76
Trp328Ser	-0.77	-0.8	-0.01	-0.03	-0.25	2	1	3	1	-0.76
Trp341Ser	-0.77	-0.8	-0.01	-0.03	-0.25	2	1	2	1	-0.76
Trp505Ser	-0.77	-0.8	-0.01	-0.03	-0.25	2	1	3	1	-0.76
Phe129Leu	-0.27	-0.3	-0.01	0.06	-0.03	2	3	4	0.75	-0.78
Phe252Leu	-0.27	-0.3	-0.01	0.06	-0.03	2	3	1	0.75	-0.78
Trp90Arg	-0.23	-0.3	0.96	0.61	-0.85	2	0	4	1	-0.87
Trp91Arg	-0.23	-0.3	0.96	0.61	-0.85	2	0	2	1	-0.87
Trp107Arg	-0.23	-0.3	0.96	0.61	-0.85	2	0	1	1	-0.87
Trp191Arg	-0.23	-0.3	0.96	0.61	-0.85	2	0	3	1	-0.87
Trp204Arg	-0.23	-0.3	0.96	0.61	-0.85	2	0	4	1	-0.87
Trp300Arg	-0.23	-0.3	0.96	0.61	-0.85	2	0	3	1	-0.87
Trp321Arg	-0.23	-0.3	0.96	0.61	-0.85	2	0	3	1	-0.87
Trp505Arg	-0.23	-0.3	0.96	0.61	-0.85	2	0	3	1	-0.87
Phe567Ser	-0.47	-0.5	0.02	0.02	-0.35	2	1	3	0.75	-0.89

Table S5. Descriptors_pncA										
WT/Mt	Molecular weight	Volume	Polarity	Isoelectric point	Hydrophobicity	Residue type(WT)	Residue type(Mt)	Secondary structure	Normalize ASA	ΔΔG (Mt-WT)
Phe13Ser	-0.47	-0.5	0.02	0.02	-0.35	2	1	1	0.75	-0.89
Phe81Ser	-0.47	-0.5	0.02	0.02	-0.35	2	1	2	0.75	-0.89
Trp68Arg	-0.23	-0.3	0.96	0.61	-0.85	2	0	2	1	-0.87
Trp119Arg	-0.23	-0.3	0.96	0.61	-0.85	2	0	1	1	-0.87
Phe58Leu	-0.27	-0.3	-0.01	0.06	-0.03	2	3	2	0.75	-0.78
Phe94Leu	-0.27	-0.3	-0.01	0.06	-0.03	2	3	2	0.75	-0.78
Phe106Leu	-0.27	-0.3	-0.01	0.06	-0.03	2	3	1	0.75	-0.78
Phe80Val	-0.37	-0.4	-0.01	0.06	-0.03	2	3	3	0.75	-0.77
Phe94Pro	-0.39	-0.2	0.02	0.1	-0.27	2	3	2	0.75	-0.77
Trp68Ser	-0.77	-0.8	-0.01	-0.03	-0.25	2	1	2	1	-0.76
Trp119Ser	-0.77	-0.8	-0.01	-0.03	-0.25	2	1	1	1	-0.76
Trp68Leu	-0.57	-0.6	-0.04	0.01	0.07	2	3	2	1	-0.65
Cys14Arg	0.41	0.5	0.97	0.71	-0.72	3	0	4	0.38	-0.62
Cys72Arg	0.41	0.5	0.97	0.71	-0.72	3	0	3	0.38	-0.62
Cys138Arg	0.41	0.5	0.97	0.71	-0.72	3	0	1	0.38	-0.62
Trp68Gly	-1	-1	-0.04	0.01	-0.08	2	3	2	1	-0.59
Cys138Ser	-0.13	0	0	0.07	-0.12	3	1	1	0.38	-0.51
Tyr34Ser	-0.59	-0.6	0	0	-0.11	2	1	4	0.83	-0.45
Tyr103Ser	-0.59	-0.6	0	0	-0.11	2	1	3	0.83	-0.45
His51Arg	0.15	0.1	0.01	0.4	-0.54	2	0	2	0.64	-0.44
His71Arg	0.15	0.1	0.01	0.4	-0.54	2	0	3	0.64	-0.44
His82Arg	0.15	0.1	0.01	0.4	-0.54	2	0	4	0.64	-0.44
His137Arg	0.15	0.1	0.01	0.4	-0.54	2	0	4	0.64	-0.44
Phe81Cys	-0.34	-0.5	0.02	-0.05	-0.23	2	3	2	0.75	-0.38
Phe94Cys	-0.34	-0.5	0.02	-0.05	-0.23	2	3	2	0.75	-0.38
Tyr64Asp	-0.37	-0.4	0.93	-0.36	0.17	2	0	4	0.83	-0.34
Tyr103Asp	-0.37	-0.4	0.93	-0.36	0.17	2	0	3	0.83	-0.34
His51Asn	-0.18	-0.2	-0.92	-0.27	-0.09	2	1	2	0.64	-0.3
Gly108Arg	0.77	0.7	1	0.6	-0.77	3	0	3	0	-0.28
Met1Thr	-0.23	-0.1	0	-0.01	-0.18	3	1	3	0.75	-0.27
His51Gln	-0.07	-0.1	-0.92	-0.24	-0.11	2	1	2	0.64	-0.27
His71Gln	-0.07	-0.1	-0.92	-0.24	-0.11	2	1	3	0.64	-0.27
Met75Thr	-0.23	-0.1	0	-0.01	-0.18	3	1	4	0.75	-0.27
Met175Thr	-0.23	-0.1	0	-0.01	-0.18	3	1	1	0.75	-0.27
Trp119Cys	-0.64	-0.8	-0.01	-0.1	-0.13	2	3	1	1	-0.25
His71Glu	-0.06	-0.1	-0.03	-0.29	-0.08	2	0	3	0.64	-0.24
Pro62Arg	0.46	0.4	0.97	0.56	-0.68	3	0	4	0.39	-0.23
Pro69Arg	0.46	0.4	0.97	0.56	-0.68	3	0	3	0.39	-0.23
Leu19Arg	0.34	0.3	1	0.6	-0.92	3	0	4	0.65	-0.22
Leu27Arg	0.34	0.3	1	0.6	-0.92	3	0	1	0.65	-0.22
Leu35Arg	0.34	0.3	1	0.6	-0.92	3	0	4	0.65	-0.22

His57Asp	-0.17	-0.2	-0.03	-0.6	0.34	2	0	1	0.64	-0.22
His57Leu	-0.19	-0.2	-0.99	-0.2	0.38	2	3	1	0.64	-0.22
His71Asp	-0.17	-0.2	-0.03	-0.6	0.34	2	0	3	0.64	-0.22
His82Asp	-0.17	-0.2	-0.03	-0.6	0.34	2	0	4	0.64	-0.22
His82Leu	-0.19	-0.2	-0.99	-0.2	0.38	2	3	4	0.64	-0.22
Leu85Arg	0.34	0.3	1	0.6	-0.92	3	0	4	0.65	-0.22
Leu116Arg	0.34	0.3	1	0.6	-0.92	3	0	1	0.65	-0.22
Leu120Arg	0.34	0.3	1	0.6	-0.92	3	0	1	0.65	-0.22
Leu159Arg	0.34	0.3	1	0.6	-0.92	3	0	2	0.65	-0.22
Met1Ile	-0.14	0	-0.03	0.04	0.19	3	3	3	0.75	-0.21
His43Pro	-0.31	-0.1	-0.96	-0.16	0.14	2	3	2	0.64	-0.21
His51Pro	-0.31	-0.1	-0.96	-0.16	0.14	2	3	2	0.64	-0.21
His57Pro	-0.31	-0.1	-0.96	-0.16	0.14	2	3	1	0.64	-0.21
His137Pro	-0.31	-0.1	-0.96	-0.16	0.14	2	3	4	0.64	-0.21
Met175Val	-0.24	-0.1	-0.03	0.03	0.11	3	3	1	0.75	-0.2
Cys14His	0.26	0.4	0.96	0.31	-0.18	3	2	4	0.38	-0.18
Gln10Arg	0.22	0.2	0.93	0.64	-0.43	1	0	2	0.62	-0.17
Gly17Ser	0.23	0.2	0.03	-0.04	-0.17	3	1	4	0	-0.17
Gly78Ser	0.23	0.2	0.03	-0.04	-0.17	3	1	1	0	-0.17
Gly97Ser	0.23	0.2	0.03	-0.04	-0.17	3	1	4	0	-0.17
Gly132Ser	0.23	0.2	0.03	-0.04	-0.17	3	1	2	0	-0.17
Lys96Arg	0.22	0.2	0.05	0.13	-0.26	0	0	2	0.78	-0.14
Gly107Lys	0.55	0.5	0.95	0.47	-0.51	3	0	1	0	-0.14
Val9Ser	-0.1	-0.1	0.03	-0.04	-0.32	3	1	4	0.46	-0.12
Tyr41His	-0.2	-0.2	0.96	0.24	-0.17	2	2	4	0.83	-0.12
Pro54Ser	-0.08	-0.1	0	-0.08	-0.08	3	1	4	0.39	-0.12
Tyr103His	-0.2	-0.2	0.96	0.24	-0.17	2	2	3	0.83	-0.12
Leu4Ser	-0.2	-0.2	0.03	-0.04	-0.32	3	1	2	0.65	-0.11
Ile5Ser	-0.2	-0.2	0.03	-0.05	-0.4	3	1	2	0.66	-0.11
Ile90Ser	-0.2	-0.2	0.03	-0.05	-0.4	3	1	4	0.66	-0.11
Ser104Arg	0.54	0.5	0.97	0.64	-0.6	1	0	3	0.24	-0.11
Gly108Gln	0.55	0.5	0.07	-0.04	-0.34	3	1	3	0	-0.11
Leu151Ser	-0.2	-0.2	0.03	-0.04	-0.32	3	1	3	0.65	-0.11
Ser179Arg	0.54	0.5	0.97	0.64	-0.6	1	0	3	0.24	-0.11
Leu182Ser	-0.2	-0.2	0.03	-0.04	-0.32	3	1	2	0.65	-0.11
Asp8Asn	-0.01	0	-0.89	0.33	-0.43	0	1	4	0.4	-0.08
Asp12Asn	-0.01	0	-0.89	0.33	-0.43	0	1	1	0.4	-0.08
Asp53Asn	-0.01	0	-0.89	0.33	-0.43	0	1	4	0.4	-0.08
Ala102Thr	0.23	0.2	0.03	-0.04	-0.18	3	1	3	0.19	-0.08
Ile133Asn	0.01	0	0.07	-0.08	-0.55	3	1	3	0.66	-0.08
Asp136Asn	-0.01	0	-0.89	0.33	-0.43	0	1	4	0.4	-0.08
Ala143Thr	0.23	0.2	0.03	-0.04	-0.18	3	1	1	0.19	-0.08
Ala146Thr	0.23	0.2	0.03	-0.04	-0.18	3	1	1	0.19	-0.08
Ala171Thr	0.23	0.2	0.03	-0.04	-0.18	3	1	1	0.19	-0.08
Ala3Gln	0.44	0.4	0.07	-0.04	-0.38	3	1	2	0.19	-0.07
Ala46Gln	0.44	0.4	0.07	-0.04	-0.38	3	1	2	0.19	-0.07
Pro54Thr	0.03	0	0	-0.08	-0.05	3	1	4	0.39	-0.07

Ile6Thr	-0.09	-0.1	0.03	-0.05	-0.37	3	1	2	0.66	-0.06
Cys14Tyr	0.46	0.6	0	0.07	-0.01	3	2	4	0.38	-0.06
Gly17Asp	0.45	0.4	0.96	-0.4	0.11	3	0	4	0	-0.06
Gly24Asp	0.45	0.4	0.96	-0.4	0.11	3	0	1	0	-0.06
Pro54Gln	0.24	0.2	0.04	-0.08	-0.25	3	1	4	0.39	-0.06
Cys72Tyr	0.46	0.6	0	0.07	-0.01	3	2	3	0.38	-0.06
Gly78Asp	0.45	0.4	0.96	-0.4	0.11	3	0	1	0	-0.06
Gly97Asp	0.45	0.4	0.96	-0.4	0.11	3	0	4	0	-0.06
Gly105Asp	0.45	0.4	0.96	-0.4	0.11	3	0	1	0	-0.06
Gly132Asp	0.45	0.4	0.96	-0.4	0.11	3	0	2	0	-0.06
Ile133Thr	-0.09	-0.1	0.03	-0.05	-0.37	3	1	3	0.66	-0.06
Cys138Tyr	0.46	0.6	0	0.07	-0.01	3	2	1	0.38	-0.06
Gly162Asp	0.45	0.4	0.96	-0.4	0.11	3	0	3	0	-0.06
Asp8Gln	0.1	0.1	-0.89	0.36	-0.45	0	1	4	0.4	-0.05
Asp12Gln	0.1	0.1	-0.89	0.36	-0.45	0	1	1	0.4	-0.05
Gly17Val	0.33	0.3	0	0	0.15	3	3	4	0	-0.05
Gly23Val	0.33	0.3	0	0	0.15	3	3	4	0	-0.05
Thr47Ser	-0.11	-0.1	0	0	-0.03	1	1	2	0.37	-0.05
Gly132Val	0.33	0.3	0	0	0.15	3	3	2	0	-0.05
Thr135Ser	-0.11	-0.1	0	0	-0.03	1	1	4	0.37	-0.05
Ala3Glu	0.45	0.4	0.96	-0.09	-0.35	3	0	2	0.19	-0.04
Ala25Glu	0.45	0.4	0.96	-0.09	-0.35	3	0	1	0.19	-0.04
Ala46Glu	0.45	0.4	0.96	-0.09	-0.35	3	0	2	0.19	-0.04
Ala171Glu	0.45	0.4	0.96	-0.09	-0.35	3	0	1	0.19	-0.04
Gln10Lys	0	0	0.88	0.51	-0.17	1	0	2	0.62	-0.03
Asp8Glu	0.11	0.1	0	0.31	-0.42	0	0	4	0.4	-0.02
Ala28Asp	0.34	0.3	0.96	-0.4	0.07	3	0	1	0.19	-0.02
Thr142Lys	0.21	0.2	0.92	0.51	-0.37	1	0	1	0.37	-0.02
Thr153Asn	0.1	0.1	0.04	-0.03	-0.18	1	1	2	0.37	-0.02
Thr168Asn	0.1	0.1	0.04	-0.03	-0.18	1	1	1	0.37	-0.02
Ala3Pro	0.2	0.4	0.03	0.04	-0.13	3	3	2	0.19	-0.01
Val7Asp	0.12	0.1	0.96	-0.4	-0.04	3	0	2	0.46	-0.01
Val7Ile	0.1	0.1	0	0.01	0.08	3	3	2	0.46	-0.01
Val9Leu	0.1	0.1	0	0	0	3	3	4	0.46	-0.01
Ala46Pro	0.2	0.4	0.03	0.04	-0.13	3	3	2	0.19	-0.01
Ala46Val	0.22	0.2	0	0	0.11	3	3	2	0.19	-0.01
Pro54Leu	0.12	0.1	-0.03	-0.04	0.24	3	3	4	0.39	-0.01
Pro62Leu	0.12	0.1	-0.03	-0.04	0.24	3	3	4	0.39	-0.01
Pro69Leu	0.12	0.1	-0.03	-0.04	0.24	3	3	3	0.39	-0.01
Val73Asp	0.12	0.1	0.96	-0.4	-0.04	3	0	2	0.46	-0.01
Ala102Val	0.22	0.2	0	0	0.11	3	3	3	0.19	-0.01
Ala102Pro	0.2	0.4	0.03	0.04	-0.13	3	3	3	0.19	-0.01
Val125Asp	0.12	0.1	0.96	-0.4	-0.04	3	0	3	0.46	-0.01
Ala134Val	0.22	0.2	0	0	0.11	3	3	4	0.19	-0.01
Val139Leu	0.1	0.1	0	0	0	3	3	1	0.46	-0.01
Ala146Val	0.22	0.2	0	0	0.11	3	3	1	0.19	-0.01
Ala146Pro	0.2	0.4	0.03	0.04	-0.13	3	3	1	0.19	-0.01

Ala161Pro	0.2	0.4	0.03	0.04	-0.13	3	3	3	0.19	-0.01
Ala171Pro	0.2	0.4	0.03	0.04	-0.13	3	3	1	0.19	-0.01
Ala171Val	0.22	0.2	0	0	0.11	3	3	1	0.19	-0.01
Val180Leu	0.1	0.1	0	0	0	3	3	3	0.46	-0.01
Ile6Leu	0	0	0	-0.01	-0.08	3	3	2	0.66	0
Lys96Asn	-0.11	-0.1	-0.88	-0.54	0.19	0	1	2	0.78	0
Val7Ala	-0.22	-0.2	0	0	-0.11	3	3	2	0.46	0.01
Val9Ala	-0.22	-0.2	0	0	-0.11	3	3	4	0.46	0.01
Leu19Pro	-0.12	0.1	0.03	0.04	-0.24	3	3	4	0.65	0.01
Leu27Pro	-0.12	0.1	0.03	0.04	-0.24	3	3	1	0.65	0.01
Leu35Pro	-0.12	0.1	0.03	0.04	-0.24	3	3	4	0.65	0.01
Asp49Val	-0.12	-0.1	-0.96	0.4	0.04	0	3	2	0.4	0.01
Leu85Pro	-0.12	0.1	0.03	0.04	-0.24	3	3	4	0.65	0.01
Val93Ala	-0.22	-0.2	0	0	-0.11	3	3	2	0.46	0.01
Leu116Val	-0.1	-0.1	0	0	0	3	3	1	0.65	0.01
Leu116Pro	-0.12	0.1	0.03	0.04	-0.24	3	3	1	0.65	0.01
Leu120Pro	-0.12	0.1	0.03	0.04	-0.24	3	3	1	0.65	0.01
Val139Ala	-0.22	-0.2	0	0	-0.11	3	3	1	0.46	0.01
Val155Ala	-0.22	-0.2	0	0	-0.11	3	3	2	0.46	0.01
Leu156Pro	-0.12	0.1	0.03	0.04	-0.24	3	3	2	0.65	0.01
Leu159Pro	-0.12	0.1	0.03	0.04	-0.24	3	3	2	0.65	0.01
Leu172Pro	-0.12	0.1	0.03	0.04	-0.24	3	3	1	0.65	0.01
Val180Ala	-0.22	-0.2	0	0	-0.11	3	3	3	0.46	0.01
Asp12Ala	-0.34	-0.3	-0.96	0.4	-0.07	0	3	1	0.4	0.02
Asp49Ala	-0.34	-0.3	-0.96	0.4	-0.07	0	3	2	0.4	0.02
Asp53Ala	-0.34	-0.3	-0.96	0.4	-0.07	0	3	4	0.4	0.02
Asp63Ala	-0.34	-0.3	-0.96	0.4	-0.07	0	3	4	0.4	0.02
Lys96Thr	-0.21	-0.2	-0.92	-0.51	0.37	0	1	2	0.78	0.02
Asn118Thr	-0.1	-0.1	-0.04	0.03	0.18	1	1	1	0.42	0.02
Leu172Ala	-0.32	-0.3	0	0	-0.11	3	3	1	0.65	0.02
Glu181Asp	-0.11	-0.1	0	-0.31	0.42	0	0	2	0.59	0.02
Lys48Gln	0	0	-0.88	-0.51	0.17	0	1	2	0.78	0.03
Lys96Gln	0	0	-0.88	-0.51	0.17	0	1	2	0.78	0.03
Ala26Gly	-0.11	-0.1	0	0	-0.04	3	3	1	0.19	0.04
Ala79Gly	-0.11	-0.1	0	0	-0.04	3	3	1	0.19	0.04
Ala102Gly	-0.11	-0.1	0	0	-0.04	3	3	3	0.19	0.04
Val7Gly	-0.33	-0.3	0	0	-0.15	3	3	2	0.46	0.05
Val9Gly	-0.33	-0.3	0	0	-0.15	3	3	4	0.46	0.05
Val21Gly	-0.33	-0.3	0	0	-0.15	3	3	4	0.46	0.05
Val44Gly	-0.33	-0.3	0	0	-0.15	3	3	2	0.46	0.05
Val45Gly	-0.33	-0.3	0	0	-0.15	3	3	2	0.46	0.05
Val125Gly	-0.33	-0.3	0	0	-0.15	3	3	3	0.46	0.05
Val128Gly	-0.33	-0.3	0	0	-0.15	3	3	2	0.46	0.05
Val130Gly	-0.33	-0.3	0	0	-0.15	3	3	2	0.46	0.05
Val139Gly	-0.33	-0.3	0	0	-0.15	3	3	1	0.46	0.05
Val155Gly	-0.33	-0.3	0	0	-0.15	3	3	2	0.46	0.05
Val180Gly	-0.33	-0.3	0	0	-0.15	3	3	3	0.46	0.05

Ser185Thr	0.11	0.1	0	0	0.03	1	1	3	0.24	0.05
Asp8Gly	-0.45	-0.4	-0.96	0.4	-0.11	0	3	4	0.4	0.06
Gln10Pro	-0.24	0	-0.04	0.08	0.25	1	3	2	0.62	0.06
Asp12Gly	-0.45	-0.4	-0.96	0.4	-0.11	0	3	1	0.4	0.06
Asp49Gly	-0.45	-0.4	-0.96	0.4	-0.11	0	3	2	0.4	0.06
Asp63Gly	-0.45	-0.4	-0.96	0.4	-0.11	0	3	4	0.4	0.06
Thr76Ile	0.09	0.1	-0.03	0.05	0.37	1	3	4	0.37	0.06
Lys96Glu	0.01	0	0.01	-0.56	0.2	0	0	2	0.78	0.06
Tyr103Cys	-0.46	-0.6	0	-0.07	0.01	2	3	3	0.83	0.06
Asp136Gly	-0.45	-0.4	-0.96	0.4	-0.11	0	3	4	0.4	0.06
Gln141Pro	-0.24	0	-0.04	0.08	0.25	1	3	1	0.62	0.06
Thr168Ile	0.09	0.1	-0.03	0.05	0.37	1	3	1	0.37	0.06
Thr47Pro	-0.03	0.2	0	0.08	0.05	1	3	2	0.37	0.07
Thr61Pro	-0.03	0.2	0	0.08	0.05	1	3	4	0.37	0.07
Thr76Pro	-0.03	0.2	0	0.08	0.05	1	3	4	0.37	0.07
Thr100Pro	-0.03	0.2	0	0.08	0.05	1	3	4	0.37	0.07
Thr114Pro	-0.03	0.2	0	0.08	0.05	1	3	2	0.37	0.07
Thr135Pro	-0.03	0.2	0	0.08	0.05	1	3	4	0.37	0.07
Thr142Pro	-0.03	0.2	0	0.08	0.05	1	3	1	0.37	0.07
Thr160Pro	-0.03	0.2	0	0.08	0.05	1	3	2	0.37	0.07
Thr47Ala	-0.23	-0.2	-0.03	0.04	0.18	1	3	2	0.37	0.08
Thr100Ala	-0.23	-0.2	-0.03	0.04	0.18	1	3	4	0.37	0.08
Thr142Ala	-0.23	-0.2	-0.03	0.04	0.18	1	3	1	0.37	0.08
Arg140Ser	-0.54	-0.5	-0.97	-0.64	0.6	0	1	1	0.93	0.11
Arg148Ser	-0.54	-0.5	-0.97	-0.64	0.6	0	1	1	0.93	0.11
Gln173Gly	-0.55	-0.5	-0.07	0.04	0.34	1	3	1	0.62	0.11
Ser185Ile	0.2	0.2	-0.03	0.05	0.4	1	3	3	0.24	0.11
His43Tyr	0.2	0.2	-0.96	-0.24	0.17	2	2	2	0.64	0.12
His51Tyr	0.2	0.2	-0.96	-0.24	0.17	2	2	2	0.64	0.12
His57Tyr	0.2	0.2	-0.96	-0.24	0.17	2	2	1	0.64	0.12
Ser59Pro	0.08	0.3	0	0.08	0.08	1	3	4	0.24	0.12
Ser65Pro	0.08	0.3	0	0.08	0.08	1	3	4	0.24	0.12
Ser66Pro	0.08	0.3	0	0.08	0.08	1	3	4	0.24	0.12
Ser67Pro	0.08	0.3	0	0.08	0.08	1	3	4	0.24	0.12
His71Tyr	0.2	0.2	-0.96	-0.24	0.17	2	2	3	0.64	0.12
Ser74Gly	-0.23	-0.2	-0.03	0.04	0.17	1	3	4	0.24	0.17
Val139Met	0.24	0.1	0.03	-0.03	-0.11	3	3	1	0.46	0.2
Val155Met	0.24	0.1	0.03	-0.03	-0.11	3	3	2	0.46	0.2
Pro62His	0.31	0.3	0.96	0.16	-0.14	3	2	4	0.39	0.21
Asp49His	0.17	0.2	0.03	0.6	-0.34	0	2	2	0.4	0.22
Asp63His	0.17	0.2	0.03	0.6	-0.34	0	2	4	0.4	0.22
Asp136His	0.17	0.2	0.03	0.6	-0.34	0	2	4	0.4	0.22
Arg121Pro	-0.46	-0.2	-0.97	-0.56	0.68	0	3	1	0.93	0.23
Arg123Pro	-0.46	-0.2	-0.97	-0.56	0.68	0	3	3	0.93	0.23
Arg140Pro	-0.46	-0.2	-0.97	-0.56	0.68	0	3	1	0.93	0.23
Cys72Trp	0.64	0.8	0.01	0.1	0.13	3	2	3	0.38	0.25
Thr87Met	0.23	0.1	0	0.01	0.18	1	3	4	0.37	0.27

Thr142Met	0.23	0.1	0	0.01	0.18	1	3	1	0.37	0.27
Arg154Gly	-0.77	-0.7	-1	-0.6	0.77	0	3	2	0.93	0.28
Asp8Tyr	0.37	0.4	-0.93	0.36	-0.17	0	2	4	0.4	0.34
Asp129Tyr	0.37	0.4	-0.93	0.36	-0.17	0	2	2	0.4	0.34
Asp136Tyr	0.37	0.4	-0.93	0.36	-0.17	0	2	4	0.4	0.34
Arg140Cys	-0.41	-0.5	-0.97	-0.71	0.72	0	3	1	0.93	0.62
Leu4Trp	0.57	0.6	0.04	-0.01	-0.07	3	2	2	0.65	0.65
Val7Phe	0.37	0.4	0.01	-0.06	0.03	3	2	2	0.46	0.77
Val131Phe	0.37	0.4	0.01	-0.06	0.03	3	2	2	0.46	0.77
Val180Phe	0.37	0.4	0.01	-0.06	0.03	3	2	3	0.46	0.77
Arg121Trp	0.23	0.3	-0.96	-0.61	0.85	0	2	1	0.93	0.87
Ser59Phe	0.47	0.5	-0.02	-0.02	0.35	1	2	4	0.24	0.89

Table S6. Descriptors_rpoB										
WT/Mutant	Molecular weight	Volume	Polarity	Isoelectric point	Hydrophobicity	Residue type(WT)	Residue type(Mt)	Secondary structure	Normalize ASA	ΔΔG (Mt-WT)
Phe101Ser	-0.47	-0.5	0.02	0.02	-0.35	2	1	2	0.81	-0.89
Phe503Ser	-0.47	-0.5	0.02	0.02	-0.35	2	1	4	0.81	-0.89
Phe505Ser	-0.47	-0.5	0.02	0.02	-0.35	2	1	3	0.81	-0.89
Phe424Leu	-0.27	-0.3	-0.01	0.06	-0.03	2	3	1	0.81	-0.78
Phe505Leu	-0.27	-0.3	-0.01	0.06	-0.03	2	3	3	0.81	-0.78
Phe248Val	-0.37	-0.4	-0.01	0.06	-0.03	2	3	4	0.81	-0.77
His445Arg	0.15	0.1	0.01	0.4	-0.54	2	0	1	0.69	-0.44
His470Arg	0.15	0.1	0.01	0.4	-0.54	2	0	4	0.69	-0.44
His674Arg	0.15	0.1	0.01	0.4	-0.54	2	0	4	0.69	-0.44
His835Arg	0.15	0.1	0.01	0.4	-0.54	2	0	4	0.69	-0.44
His445Asn	-0.18	-0.2	-0.92	-0.27	-0.09	2	1	1	0.69	-0.3
His445Gln	-0.07	-0.1	-0.92	-0.24	-0.11	2	1	1	0.69	-0.27
Met666Thr	-0.23	-0.1	0	-0.01	-0.18	3	1	3	0.81	-0.27
Met707Thr	-0.23	-0.1	0	-0.01	-0.18	3	1	2	0.81	-0.27
Leu47Arg	0.34	0.3	1	0.6	-0.92	3	0	4	0.69	-0.22
Leu378Arg	0.34	0.3	1	0.6	-0.92	3	0	1	0.69	-0.22
Leu430Arg	0.34	0.3	1	0.6	-0.92	3	0	4	0.69	-0.22
His445Asp	-0.17	-0.2	-0.03	-0.6	0.34	2	0	1	0.69	-0.22
His445Leu	-0.19	-0.2	-0.99	-0.2	0.38	2	3	1	0.69	-0.22
His723Asp	-0.17	-0.2	-0.03	-0.6	0.34	2	0	4	0.69	-0.22
Met154Leu	-0.14	0	-0.03	0.03	0.11	3	3	2	0.81	-0.21
Met601Ile	-0.14	0	-0.03	0.04	0.19	3	3	1	0.81	-0.21
His835Pro	-0.31	-0.3	-0.96	-0.16	0.14	2	3	4	0.69	-0.21
Met434Val	-0.24	-0.1	-0.03	0.03	0.11	3	3	3	0.81	-0.2
Gly368Ser	0.23	0.2	0.03	-0.04	-0.17	3	1	4	0	-0.17
Gly456Ser	0.23	0.2	0.03	-0.04	-0.17	3	1	4	0	-0.17
Lys446Arg	0.22	0.2	0.05	0.13	-0.26	0	0	4	0.84	-0.14
Pro45Ser	-0.08	-0.1	0	-0.08	-0.08	3	1	4	0.42	-0.12
Pro280Ser	-0.08	-0.1	0	-0.08	-0.08	3	1	3	0.42	-0.12
Pro551Ser	-0.08	-0.1	0	-0.08	-0.08	3	1	4	0.42	-0.12
Ser428Arg	0.54	0.5	0.97	0.64	-0.6	1	0	4	0.26	-0.11
Ser431Arg	0.54	0.5	0.97	0.64	-0.6	1	0	2	0.26	-0.11
Asp545Asn	-0.01	0	-0.89	0.33	-0.43	0	1	4	0.44	-0.08
Pro471Thr	0.03	0	0	-0.08	-0.05	3	1	4	0.42	-0.07
Pro479Thr	0.03	0	0	-0.08	-0.05	3	1	4	0.42	-0.07
Pro962Thr	0.03	0	0	-0.08	-0.05	3	1	4	0.42	-0.07
Pro227Gln	0.24	0.2	0.04	-0.08	-0.25	3	1	3	0.42	-0.06
Ile910Thr	-0.09	-0.1	0.03	-0.05	-0.37	3	1	2	0.71	-0.06
Gly178Val	0.33	0.3	0	0	0.15	3	3	2	0	-0.05
Thr508Ser	-0.11	-0.1	0	0	-0.03	1	1	2	0.4	-0.05
Leu735Gln	0.12	0.1	0.07	-0.04	-0.49	3	1	1	0.69	-0.05

Leu1122Gln	0.12	0.1	0.07	-0.04	-0.49	3	1	1	0.69	-0.05
Asn487Ser	-0.21	-0.2	-0.04	0.03	0.15	1	1	4	0.45	-0.03
Gln606Lys	0	0	0.88	0.51	-0.17	1	0	4	0.66	-0.03
Asp103Glu	0.11	0.1	0	0.31	-0.42	0	0	2	0.44	-0.02
Asp545Glu	0.11	0.1	0	0.31	-0.42	0	0	4	0.44	-0.02
Asp574Glu	0.11	0.1	0	0.31	-0.42	0	0	2	0.44	-0.02
Ala670Asp	0.34	0.3	0.96	-0.4	0.07	3	0	2	0.21	-0.02
Pro76Leu	0.12	0.1	-0.03	-0.04	0.24	3	3	4	0.42	-0.01
Val496Leu	0.1	0.1	0	0	0	3	3	4	0.5	-0.01
Arg39Arg	0	0	0	0	0	0	0	3	1	0
Ile90Leu	0	0	0	-0.01	-0.08	3	3	2	0.71	0
Gly149Gly	0	0	0	0	0	3	3	2	0	0
Leu430Pro	-0.12	-0.1	0.03	0.04	-0.24	3	3	4	0.69	0.01
Asp435Val	-0.12	-0.1	-0.96	0.4	0.04	0	3	3	0.44	0.01
Leu452Pro	-0.12	-0.1	0.03	0.04	-0.24	3	3	2	0.69	0.01
Ile480Val	-0.1	-0.1	0	-0.01	-0.08	3	3	4	0.71	0.01
Ile488Val	-0.1	-0.1	0	-0.01	-0.08	3	3	4	0.71	0.01
Ile491Val	-0.1	-0.1	0	-0.01	-0.08	3	3	2	0.71	0.01
Val496Ala	-0.22	-0.2	0	0	-0.11	3	3	4	0.5	0.01
Val518Ala	-0.22	-0.2	0	0	-0.11	3	3	2	0.5	0.01
Leu731Pro	-0.12	-0.1	0.03	0.04	-0.24	3	3	2	0.69	0.01
Ile783Val	-0.1	-0.1	0	-0.01	-0.08	3	3	3	0.71	0.01
Leu815Val	-0.1	-0.1	0	0	0	3	3	1	0.69	0.01
Pro899Ala	-0.2	-0.2	-0.03	-0.04	0.13	3	3	3	0.42	0.01
Ile925Val	-0.1	-0.1	0	-0.01	-0.08	3	3	1	0.71	0.01
Glu356Asp	-0.11	-0.1	0	-0.31	0.42	0	0	2	0.64	0.02
Asp571Ala	-0.34	-0.3	-0.96	0.4	-0.07	0	3	3	0.44	0.02
Glu639Asp	-0.11	-0.1	0	-0.31	0.42	0	0	3	0.64	0.02
Glu761Asp	-0.11	-0.1	0	-0.31	0.42	0	0	3	0.64	0.02
Glu978Asp	-0.11	-0.1	0	-0.31	0.42	0	0	1	0.64	0.02
Glu789Val	-0.23	-0.2	-0.96	0.09	0.46	0	3	3	0.64	0.03
Ala584Gly	-0.11	-0.1	0	0	-0.04	3	3	4	0.21	0.04
Val695Gly	-0.33	-0.3	0	0	-0.15	3	3	2	0.5	0.05
Asp265Gly	-0.45	-0.4	-0.96	0.4	-0.11	0	3	1	0.44	0.06
Thr350Ile	0.09	0.1	-0.03	0.05	0.37	1	3	2	0.4	0.06
Thr361Ile	0.09	0.1	-0.03	0.05	0.37	1	3	3	0.4	0.06
Thr400Ile	0.09	0.1	-0.03	0.05	0.37	1	3	4	0.4	0.06
Gln432Pro	-0.24	-0.2	-0.04	0.08	0.25	1	3	2	0.66	0.06
Asp435Gly	-0.45	-0.4	-0.96	0.4	-0.11	0	3	3	0.44	0.06
Asp634Gly	-0.45	-0.4	-0.96	0.4	-0.11	0	3	3	0.44	0.06
Asp1006Gly	-0.45	-0.4	-0.96	0.4	-0.11	0	3	2	0.44	0.06
Thr427Pro	-0.03	0	0	0.08	0.05	1	3	4	0.4	0.07
Thr508Pro	-0.03	0	0	0.08	0.05	1	3	2	0.4	0.07
Glu82Gly	-0.56	-0.5	-0.96	0.09	0.31	0	3	1	0.64	0.08
Thr399Ala	-0.23	-0.2	-0.03	0.04	0.18	1	3	4	0.4	0.08
Glu460Gly	-0.56	-0.5	-0.96	0.09	0.31	0	3	4	0.64	0.08
Thr508Ala	-0.23	-0.2	-0.03	0.04	0.18	1	3	2	0.4	0.08

Asn658Asp	0.01	0	0.89	-0.33	0.43	1	0	4	0.45	0.08
Glu812Gly	-0.56	-0.5	-0.96	0.09	0.31	0	3	1	0.64	0.08
Thr913Ala	-0.23	-0.2	-0.03	0.04	0.18	1	3	4	0.4	0.08
Arg39Ser	-0.54	-0.5	-0.97	-0.64	0.6	0	1	3	1	0.11
Ser388Leu	0.2	0.2	-0.03	0.04	0.32	1	3	1	0.26	0.11
Ser450Leu	0.2	0.2	-0.03	0.04	0.32	1	3	2	0.26	0.11
His445Tyr	0.2	0.2	-0.96	-0.24	0.17	2	2	1	0.69	0.12
Ser450Pro	0.08	0.1	0	0.08	0.08	1	3	2	0.26	0.12
His674Tyr	0.2	0.2	-0.96	-0.24	0.17	2	2	4	0.69	0.12
His723Tyr	0.2	0.2	-0.96	-0.24	0.17	2	2	4	0.69	0.12
His745Tyr	0.2	0.2	-0.96	-0.24	0.17	2	2	2	0.69	0.12
Ser34Ala	-0.12	-0.1	-0.03	0.04	0.21	1	3	3	0.26	0.13
Ser1124Ala	-0.12	-0.1	-0.03	0.04	0.21	1	3	1	0.26	0.13
Ser431Gly	-0.23	-0.2	-0.03	0.04	0.17	1	3	2	0.26	0.17
Val496Met	0.24	0.1	0.03	-0.03	-0.11	3	3	4	0.5	0.2
Val513Met	0.24	0.1	0.03	-0.03	-0.11	3	3	2	0.5	0.2
Val970Met	0.24	0.1	0.03	-0.03	-0.11	3	3	4	0.5	0.2
Cys681Trp	0.64	0.8	0.01	0.1	0.13	3	2	2	0.41	0.25
Thr508His	0.28	0.3	0.96	0.24	-0.09	1	2	2	0.4	0.28
Asp435Tyr	0.37	0.4	-0.93	0.36	-0.17	0	2	3	0.44	0.34
Arg662His	-0.15	-0.1	-0.01	-0.4	0.54	0	2	2	1	0.44
Arg827Cys	-0.41	-0.5	-0.97	-0.71	0.72	0	3	2	1	0.62
Ser450Trp	0.77	0.8	0.01	0.03	0.25	1	2	2	0.26	0.76
Val146Phe	0.37	0.4	0.01	-0.06	0.03	3	2	2	0.5	0.77
Val170Phe	0.37	0.4	0.01	-0.06	0.03	3	2	3	0.5	0.77
Leu42Phe	0.27	0.3	0.01	-0.06	0.03	3	2	3	0.69	0.78

Table S7. *gyrA*							
**Uncertainity**	**Relief**	**OneR**	**InfoGain**	**GainRatio**	**Correlation**	**Classifier**	**BestFirst**
1 10 ??G (Mt-WT)	0.187037 6 Residue type(WT)	100 10 ??G (Mt-WT)	0.986 10 ??G (Mt-WT)	1 10 ??G (Mt-WT)	0.678 10 ??G (Mt-WT)	0 10 ??G (Mt-WT)	Molecular weight
0.26 3 Polarity	0.163426 7 Residue type(Mt)	79.167 7 Residue type(Mt)	0.248 7 Residue type(Mt)	0.293 3 Polarity	0.529 6 Residue type(WT)	0 3 Polarity	Polarity
0.254 7 Residue type(Mt)	0.082888 10 ??G (Mt-WT)	77.778 6 Residue type(WT)	0.238 6 Residue type(WT)	0.257 7 Residue type(Mt)	0.465 7 Residue type(Mt)	0 2 Volume	Residue type(Mt)
0.24 6 Residue type(WT)	0.074167 9 Normalize ASA	73.611 4 Isoelectric point	0.232 4 Isoelectric point	0.251 9 Normalize ASA	0.434 5 Hydrophobicity	0 9 Normalize ASA	Normalize ASA
0.223 9 Normalize ASA	0.060634 4 Isoelectric point	72.222 2 Volume	0.231 3 Polarity	0.238 6 Residue type(WT)	0.417 1 Molecular weight	0 4 Isoelectric point	??G (Mt-WT)
0.205 1 Molecular weight	0.046162 5 Hydrophobicity	72.222 1 Molecular weight	0.201 1 Molecular weight	0.225 5 Hydrophobicity	0.397 2 Volume	0 5 Hydrophobicity	
0.193 4 Isoelectric point	0.038153 3 Polarity	70.833 9 Normalize ASA	0.198 9 Normalize ASA	0.206 1 Molecular weight	0.39 9 Normalize ASA	0 6 Residue type(WT)	
0.173 5 Hydrophobicity	0.03631 2 Volume	65.278 5 Hydrophobicity	0.146 2 Volume	0.164 4 Isoelectric point	0.28 3 Polarity	0 7 Residue type(Mt)	
0.148 2 Volume	0.035281 1 Molecular weight	58.333 3 Polarity	0.139 5 Hydrophobicity	0.148 2 Volume	0.174 8 Secondary structure	0 8 Secondary structure	
0 8 Secondary structure	-0.000463 8 Secondary structure	58.333 8 Secondary structure	0 8 Secondary structure	0 8 Secondary structure	0.106 4 Isoelectric point	0 1 Molecular weight	

Table S8. *gyrB*							
**Uncertainity**	**Relief**	**OneR**	**InfoGain**	**GainRatio**	**Correlation**	**Classifier**	**BestFirst**
1 10 ??G (Mt-WT)	0.38 6 Residue type(WT)	100 10 ??G (Mt-WT)	0.985 10 ??G (Mt-WT)	1 10 ??G (Mt-WT)	0.6234 6 Residue type(WT)	0 10 ??G (Mt-WT)	Volume
0.415 6 Residue type(WT)	0.11 10 ??G (Mt-WT)	83.673 6 Residue type(WT)	0.411 6 Residue type(WT)	0.414 6 Residue type(WT)	0.6097 10 ??G (Mt-WT)	0 3 Polarity	Isoelectric point
0.329 2 Volume	0.10 4 Isoelectric point	83.673 2 Volume	0.374 2 Volume	0.36 4 Isoelectric point	0.5388 5 Hydrophobicity	0 2 Volume	Hydrophobicity
0.308 5 Hydrophobicity	0.09 5 Hydrophobicity	81.633 5 Hydrophobicity	0.305 5 Hydrophobicity	0.307 5 Hydrophobicity	0.4139 4 Isoelectric point	0 9 Normalize ASA	Residue type(WT)
0.284 4 Isoelectric point	0.07 7 Residue type(Mt)	75.51 1 Molecular weight	0.24 9 Normalize ASA	0.299 9 Normalize ASA	0.3897 3 Polarity	0 4 Isoelectric point	??G (Mt-WT)
0.269 9 Normalize ASA	0.04 2 Volume	67.347 4 Isoelectric point	0.231 4 Isoelectric point	0.291 2 Volume	0.3878 9 Normalize ASA	0 5 Hydrophobicity	
0 8 Secondary structure	0.04 1 Molecular weight	67.347 7 Residue type(Mt)	0 8 Secondary structure	0 7 Residue type(Mt)	0.2436 8 Secondary structure	0 6 Residue type(WT)	
0 7 Residue type(Mt)	0.03 9 Normalize ASA	65.306 9 Normalize ASA	0 7 Residue type(Mt)	0 3 Polarity	0.1642 1 Molecular weight	0 7 Residue type(Mt)	
0 3 Polarity	0.01 3 Polarity	65.306 8 Secondary structure	0 3 Polarity	0 8 Secondary structure	0.1307 2 Volume	0 8 Secondary structure	
0 1 Molecular weight	0.00 8 Secondary structure	63.265 3 Polarity	0 1 Molecular weight	0 1 Molecular weight	0.0267 7 Residue type(Mt)	0 1 Molecular weight	

Table S9. *inhA*							
**Uncertainity**	**Relief**	**OneR**	**InfoGain**	**GainRatio**	**Correlation**	**Classifier**	**BestFirst**
1 10 ??G (Mt-WT)	0.4218 7 Residue type(Mt)	100 10 ??G (Mt-WT)	0.951 10 ??G (Mt-WT)	1 10 ??G (Mt-WT)	0.6987 10 ??G (Mt-WT)	0 10 ??G (Mt-WT)	Hydrophobicity
0.667 5 Hydrophobicity	0.2249 5 Hydrophobicity	85.1852 7 Residue type(Mt)	0.609 5 Hydrophobicity	0.695 5 Hydrophobicity	0.6721 7 Residue type(Mt)	0 3 Polarity	Residue type(Mt)
0.516 7 Residue type(Mt)	0.1476 10 ??G (Mt-WT)	85.1852 3 Polarity	0.503 7 Residue type(Mt)	0.504 7 Residue type(Mt)	0.6361 5 Hydrophobicity	0 2 Volume	??G (Mt-WT)
0.406 3 Polarity	0.0558 4 Isoelectric point	81.4815 5 Hydrophobicity	0.394 3 Polarity	0.398 3 Polarity	0.4203 3 Polarity	0 9 Normalize ASA	
0 8 Secondary structure	0.0558 3 Polarity	74.0741 9 Normalize ASA	0 8 Secondary structure	0 8 Secondary structure	0.292 8 Secondary structure	0 4 Isoelectric point	
0 2 Volume	0.0429 6 Residue type(WT)	70.3704 4 Isoelectric point	0 2 Volume	0 2 Volume	0.2559 6 Residue type(WT)	0 5 Hydrophobicity	
0 4 Isoelectric point	0.0217 8 Secondary structure	66.6667 2 Volume	0 4 Isoelectric point	0 4 Isoelectric point	0.2042 4 Isoelectric point	0 6 Residue type(WT)	
0 9 Normalize ASA	0.0216 2 Volume	62.963 6 Residue type(WT)	0 9 Normalize ASA	0 9 Normalize ASA	0.1948 1 Molecular weight	0 7 Residue type(Mt)	
0 6 Residue type(WT)	0.016 1 Molecular weight	55.5556 1 Molecular weight	0 6 Residue type(WT)	0 6 Residue type(WT)	0.1572 2 Volume	0 8 Secondary structure	
0 1 Molecular weight	-0.0127 9 Normalize ASA	48.1481 8 Secondary structure	0 1 Molecular weight	0 1 Molecular weight	0.0166 9 Normalize ASA	0 1 Molecular weight	

Table S10. *katG*							
**Uncertainity**	**Relief**	**OneR**	**InfoGain**	**GainRatio**	**Correlation**	**Classifier**	**BestFirst**
1 10 ??G	0.1836 7 Residue type(Mt)	100 10 ??G	0.995 10 ??G	1 10 ??G	0.617 10 ??G	0 10 ??G	Polarity
0.232 5 Hydrophobicity	0.1815 6 Residue type(WT)	81.6 4 Isoelectric point	0.375 5 Hydrophobicity	0.2269 6 Residue type(WT)	0.475 5 Hydrophobicity	0 3 Polarity	Hydrophobicity
0.225 6 Residue type(WT)	0.1231 10 ??G	79.2 9 Normalize ASA	0.31 9 Normalize ASA	0.2016 3 Polarity	0.472 6 Residue type(WT)	0 2 Volume	Residue type(WT)
0.224 9 Normalize ASA	0.0939 9 Normalize ASA	77.2 6 Residue type(WT)	0.224 3 Polarity	0.1747 9 Normalize ASA	0.441 7 Residue type(Mt)	0 9 Normalize ASA	Residue type(Mt)
0.213 3 Polarity	0.075 5 Hydrophobicity	77.2 5 Hydrophobicity	0.223 4 Isoelectric point	0.1679 5 Hydrophobicity	0.4 3 Polarity	0 4 Isoelectric point	??G
0.183 7 Residue type(Mt)	0.0724 3 Polarity	74 7 Residue type(Mt)	0.221 6 Residue type(WT)	0.1614 7 Residue type(Mt)	0.18 1 Molecular weight	0 5 Hydrophobicity	
0.17 4 Isoelectric point	0.0677 4 Isoelectric point	73.6 1 Molecular weight	0.212 7 Residue type(Mt)	0.1372 4 Isoelectric point	0.166 4 Isoelectric point	0 6 Residue type(WT)	
0.138 1 Molecular weight	0.0487 2 Volume	69.6 3 Polarity	0.181 1 Molecular weight	0.1114 1 Molecular weight	0.15 2 Volume	0 7 Residue type(Mt)	
0.101 2 Volume	0.0452 1 Molecular weight	68 2 Volume	0.114 2 Volume	0.0894 2 Volume	0.146 8 Secondary structure	0 8 Secondary structure	
0 8 Secondary structure	-0.0345 8 Secondary structure	58.8 8 Secondary structure	0 8 Secondary structure	0 8 Secondary structure	0.125 9 Normalize ASA	0 1 Molecular weight	

Table S11. *pncA*							
**Uncertainity**	**Relief**	**OneR**	**InfoGain**	**GainRatio**	**Correlation**	**Classifier**	**BestFirst**
1 10 ??G	0.2542 7 Residue type(Mt)	100 10 ??G	0.9829 10 ??G	1 10 ??G	0.6148 10 ??G	0.24481 10 ??G	Molecular weight
0.2708 7 Residue type(Mt)	0.132 6 Residue type(WT)	81.328 5 Hydrophobicity	0.2666 7 Residue type(Mt)	0.2703 7 Residue type(Mt)	0.5453 7 Residue type(Mt)	0.16598 6 Residue type(WT)	Residue type(WT)
0.2174 5 Hydrophobicity	0.1058 10 ??G	77.178 7 Residue type(Mt)	0.2071 5 Hydrophobicity	0.2246 5 Hydrophobicity	0.3902 3 Polarity	0.13444 7 Residue type(Mt)	Residue type(Mt)
0.1711 3 Polarity	0.0891 9 Normalize ASA	75.104 4 Isoelectric point	0.1887 3 Polarity	0.1544 3 Polarity	0.3894 5 Hydrophobicity	0.05809 3 Polarity	??G
0.1523 6 Residue type(WT)	0.0728 3 Polarity	74.274 6 Residue type(WT)	0.1865 6 Residue type(WT)	0.1272 6 Residue type(WT)	0.3822 6 Residue type(WT)	0.05602 5 Hydrophobicity	
0.1154 9 Normalize ASA	0.0677 5 Hydrophobicity	74.274 9 Normalize ASA	0.1426 9 Normalize ASA	0.0958 9 Normalize ASA	0.2486 1 Molecular weight	0 9 Normalize ASA	
0.0874 1 Molecular weight	0.0662 4 Isoelectric point	68.465 3 Polarity	0.0861 1 Molecular weight	0.0872 1 Molecular weight	0.1459 2 Volume	0 2 Volume	
0 2 Volume	0.0571 1 Molecular weight	67.635 1 Molecular weight	0 2 Volume	0 2 Volume	0.0937 4 Isoelectric point	0 8 Secondary structure	
0 8 Secondary structure	0.0494 2 Volume	57.676 2 Volume	0 8 Secondary structure	0 8 Secondary structure	0.0824 9 Normalize ASA	0 4 Isoelectric point	
0 4 Isoelectric point	-0.0766 8 Secondary structure	55.602 8 Secondary structure	0 4 Isoelectric point	0 4 Isoelectric point	0.0271 8 Secondary structure	-0.00415 1 Molecular weight	

Table S12. *rpoB*						
**Relief**	**OneR**	**InfoGain**	**GainRatio**	**Correlation**	**Classifier**	**BestFirst**
0.2684 7 Residue type(Mt)	100 10 ??G (Mt-WT)	0.9891 10 ??G (Mt-WT)	1 10 ??G (Mt-WT)	0.6388 10 ??G (Mt-WT)	0 10 ??G (Mt-WT)	Hydrophobicity
0.1254 6 Residue type(WT)	84.211 7 Residue type(Mt)	0.3897 7 Residue type(Mt)	0.375 5 Hydrophobicity	0.5697 7 Residue type(Mt)	0 3 Polarity	Residue type(WT)
0.1083 10 ??G (Mt-WT)	72.807 5 Hydrophobicity	0.2587 5 Hydrophobicity	0.284 7 Residue type(Mt)	0.5034 5 Hydrophobicity	0 2 Volume	Residue type(Mt)
0.061 5 Hydrophobicity	71.93 9 Normalize ASA	0.1096 6 Residue type(WT)	0.115 3 Polarity	0.2905 6 Residue type(WT)	0 9 Normalize ASA	??G (Mt-WT)
0.0443 9 Normalize ASA	63.158 1 Molecular weight	0.0749 3 Polarity	0.112 6 Residue type(WT)	0.2794 3 Polarity	0 4 Isoelectric point	
0.0432 4 Isoelectric point	63.158 4 Isoelectric point	0 8 Secondary structure	0 2 Volume	0.1507 4 Isoelectric point	0 5 Hydrophobicity	
0.0284 3 Polarity	61.404 8 Secondary structure	0 2 Volume	0 8 Secondary structure	0.1354 8 Secondary structure	0 6 Residue type(WT)	
0.0257 2 Volume	58.772 6 Residue type(WT)	0 4 Isoelectric point	0 4 Isoelectric point	0.1173 1 Molecular weight	0 7 Residue type(Mt)	
0.0252 1 Molecular weight	56.14 3 Polarity	0 9 Normalize ASA	0 9 Normalize ASA	0.0897 9 Normalize ASA	0 8 Secondary structure	
-0.0319 8 Secondary structure	53.509 2 Volume	0 1 Molecular weight	0 1 Molecular weight	0.0793 2 Volume	0 1 Molecular weight	
